# A *Filifactor alocis*-centered co-occurrence group associates with periodontitis across different oral habitats

**DOI:** 10.1038/srep09053

**Published:** 2015-03-12

**Authors:** Hui Chen, Ying Liu, Menghui Zhang, Guoyang Wang, Zhengnan Qi, Laura Bridgewater, Liping Zhao, Zisheng Tang, Xiaoyan Pang

**Affiliations:** 1 State Key Laboratory of Microbial Metabolism, School of Life Sciences and Biotechnology, Shanghai Jiao Tong University, Shanghai 200240, China; 2 Department of Endodontics, Ninth People's Hospital, Shanghai Jiao Tong University School of Medicine, Shanghai Key Laboratory of Stomatology, Shanghai 200011, China; 3 Shanghai Center for Systems Biomedicine, Shanghai Jiao Tong University, Shanghai 200240, China; 4 Department of Microbiology and Molecular Biology, Brigham Young University, Provo, Utah 84602, USA

## Abstract

Periodontitis is a highly prevalent polymicrobial disease worldwide, yet the synergistic pattern of the multiple oral pathogens involved is still poorly characterized. Here, saliva, supragingival and subgingival plaque samples from periodontitis patients and periodontally healthy volunteers were collected and profiled with 16S rRNA gene pyrosequencing. Different oral habitats harbored significantly different microbiota, and segregation of microbiota composition between periodontitis and health was observed as well. Two-step redundancy analysis identified twenty-one OTUs, including *Porphyromonas gingivalis*, *Tannerella forsythia* and *Filifactor alocis*, as potential pathogens that were significantly associated with periodontitis and with two periodontitis diagnostic parameters (pocket depth and attachment loss) in both saliva and supragingival plaque habitats. Interestingly, pairwise correlation analysis among the 21 OTUs revealed that *Filifactor alocis* was positively correlated with seven other putative pathogens (R > 0.6, *P* < 0.05), forming a co-occurrence group that was remarkably enriched in all three habitats of periodontitis patients. This bacterial cluster showed a higher diagnostic value for periodontitis than did any individual potential pathogens, especially in saliva. Thus, our study identified a potential synergistic ecological pattern involving eight co-infecting pathogens across various oral habitats, providing a new framework for understanding the etiology of periodontitis and developing new diagnoses and therapies.

Periodontitis is a polymicrobial disease of the oral cavity and a risk factor for chronic diseases such as cardiovascular disease and diabetes mellitus^1^. The complex microbiota inhabiting the biofilm around teeth and gingival tissue may play a pivotal role in the development of periodontitis^1^,^2^,^3^. Cultivation^4^,^5^ and culture-independent approaches such as PCR-based assays and DNA hybridization^6^,^7^,^8^,^9^ have previously identified several microorganisms associated with the development of periodontitis and regarded as the putative pathogens, including *Porphyromonas gingivalis*, *Tannerella forsythia*, and *Treponema denticola*. More recently, the advent of high-throughput next generation sequencing techniques allowed us to characterize the overall structure of the complex oral microbial ecosystem at the OTU level, confirming the association of those potential pathogens with periodontitis and revealing additional periodontitis-associated phylotypes such as *Prevotella* spp., *Porphyromonas endodontalis*, *Filifactor alocis*, and *Peptostreptococcus stomatis*^10^,^11^,^12^,^13^,^14^,^15^.

The oral cavity is a dynamic ‘continuum with the external environment'^16^. Though saliva, supragingival and subgingival plaque maintain unique ecosystems with distinct atmospheric and nutritional environments that favor different microbial commensals^17^,^18^,^19^, the microbiota in different intraoral habitats interact with each other continuously. Dental plaque is permanently in contact with saliva. In periodontitis patients, the microbial inhabitants of saliva and supragingival plaque inoculate and colonize subgingival plaque, thus accelerating the formation of periodontal pockets, the major lesion of periodontitis. Likewise, pathogens residing in periodontal pockets spill over to the oral cavity and can be detected in saliva and supragingival plaque. Analyzing the microbiota in these environmentally distinct and yet interacting oral habitats will provide a more comprehensive understanding of the etiology and pathogenesis of periodontitis.

Polymicrobial diseases involve co-infection with multiple bacteria, which may act synergistically to contribute to disease progression and clinical outcomes^20^. Such pathogens should be more likely to coexist at the diseased lesion, thus exhibiting a co-occurrence ecological pattern. For periodontal diseases, numerous bacteriologic studies clearly support the capacity of a variety of bacterial species coexisting in a complex biofilm to contribute to periodontitis^5^,^6^,^21^. In 1998, Socransky *et al.* defined subgingival bacterial communities by checkerboard DNA-DNA hybridization and observed ecological interbacterial relationships among 32 examined species. They found that those species could be clustered into five complexes in which members showed highly similar distribution in subgingival microbiota^6^.

After that initial effort to elucidate the ecological relationships of oral microorganisms, few studies explored the inherent coexistence and distribution patterns of multiple pathogens in human oral samples *in situ*. Instead, recent studies have mainly focused on interactions between putative pathogens using *in vitro* or animal models. For instance, a rat model of periodontitis was used to study the synergistic virulence of *P. gingivalis*, *T. denticola* and *T. forsythia*, demonstrating that those three pathogens existed as a consortium and synergistically increased alveolar bone resorption in co-infected rats^22^. An *in vitro* flow cell biofilm model demonstrated that *P. gingivalis* and *T. denticola* exhibit a strong synergy in polymicrobial biofilm formation^23^. Such studies established that *P. gingivalis* can interact synergistically with other pathogens, but not whether this pathogen actually co-exists and co-infects with the other tested pathogens *in situ*. Many putative pathogens have been associated with periodontitis. Whether these bacteria contribute to periodontal disease in random combinations or via specific synergistic patterns still remains a fundamental question.

Using 16S rRNA pyrosequencing, we obtained a comprehensive view of the oral bacterial communities in saliva, supragingival plaque and subgingival plaque from Chinese adults with and without periodontitis. We identified periodontitis-associated bacterial species that are commonly shared across different oral habitats, and we detected a *Filifactor alocis*-centered co-occurrence group of potential pathogens that was significantly enriched in periodontitis samples. These results laid a microbial ecological foundation for future etiological studies in periodontitis.

## Results

### Overall structure of oral microbiota in periodontitis and health

A total of 307,679 usable raw pyrosequencing reads passing the quality control standards were obtained from 150 samples, with an average of 2051 reads per sample (see Supplementary Fig. S1a online). After alignment in the Greengenes database and CD-hit clustering with 99.9% similarity, the unique representative sequences were classified into 1889 OTUs at 96% similarity level (see Supplementary Fig. S1b online). The richness of oral bacterial communities in three oral habitats of periodontitis patients and the control cohort were estimated by rarefaction and Chao 1, and diversity was estimated by Shannon diversity index and Simpson diversity. The shape of the rarefaction curves indicated new phylotypes would be expected with additional sequencing. However, the Shannon diversity index curves of all samples reached plateaus with the current sequencing, suggesting that most diversity had already been captured (see Supplementary Fig. S2 online).

**Figure 1:**
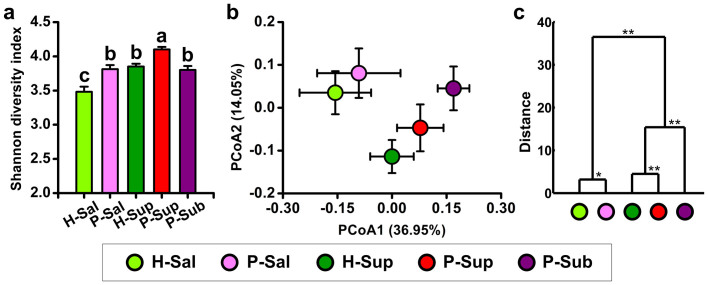
Overall structural comparison of oral microbiota. (a) Shannon diversity index of oral microbiota in different groups. Values are shown as mean ± SEM. The different letters above the columns mean significant differences among five groups as assessed by ANOVA (*P* < 0.05). (b) Principal coordinate analysis (PCoA) scores plot based on weighted UniFrac metrics. Each point represents the mean principal component scores of all subjects in a group, and the error bar represents standard deviation. (c) Clustering of oral microbiota based on distances calculated using multivariate analysis of variance (MANOVA) on the first sixteen principal coordinate scores, * *P* < 0.05; ** *P* < 0.01. The abbreviations of group names are as follows: H: Periodontally healthy volunteers; P: Periodontitis patients; Sal: Saliva; Sup: Supragingival plaque; Sub: Subgingival plaque.

As revealed by the Shannon diversity index, supragingival plaque possessed the highest level of bacterial diversity, whereas saliva showed the lowest diversity. A higher level of diversity was observed in periodontitis individuals than healthy controls in both saliva and supragingival plaque (Figure 1a). The Simpson diversity index showed a similar tendency as Shannon diversity index, and the richness of the oral microbiota estimated by rarefaction and Chao 1 displayed a similar pattern (see Supplementary Table S1 online).

Principal coordinate analysis (PCoA) based on weighted UniFrac matrices revealed remarkable differentiation of the bacterial communities in the five groups, and the differences between oral habitats were more distinct than those between patients and healthy controls of the same habitats (Figure 1b). These findings were confirmed by clustering analysis based on distances calculated using multivariate analysis of variance (MANOVA) (Figure 1c).

### Common and distinct taxa of oral microbiota in different oral habitats

Thirteen phyla with 143 genera were detected in the oral microbiota. *Bacteroidetes*, *Firmicutes*, *Proteobacteria*, *Fusobacteria*, *Actinobacteria* and *Spirochaetes* were the most predominant phyla when the three oral habitats were considered together, contributing to 39.1%, 23.2%, 13.4%, 11.4%, 8.3% and 2.1% of all reads, respectively.

Despite the high variability of oral microbiota among different oral habitats as revealed by PCoA, nine genera were present in all 150 oral samples. Those nine genera represented over half of the oral microbiome collectively (57.97% of all reads), constituting the predominant oral inhabitants. Among them, three genera-*Streptococcus*, *Leptotrichia* and *Actinomyces* accounting for 18.90% of all reads-were stable in subjects with different periodontal health status, as revealed by the similar relative abundances in saliva and supragingival plaque from periodontitis and healthy cohorts. These three genera represent the core members of the oral microbiota. The remaining six genera, including *Prevotella*, *Porphyromonas*, *Capnocytophaga*, *Fusobacterium*, *TM7_genera_incertae_sedis* and *Clostridium XIVa* displayed significantly different abundance (Mann-Whitney U test, *P* < 0.05) between health and periodontitis (see Supplementary Table S2 online).

At the OTU level, three OTUs were present in all 150 samples. Two of them (OTU0039 and OTU0202), which were closely related to *Streptococcus* sp. and *Leptotrichia buccalis*, showed similar relative abundance between periodontitis patients and healthy controls in saliva and supragingival plaque. The third OTU (OTU0288), which was highly homologous with *Prevotella* sp. showed different abundance between periodontitis and healthy samples (see Supplementary Table S2 online).

**Figure 2:**
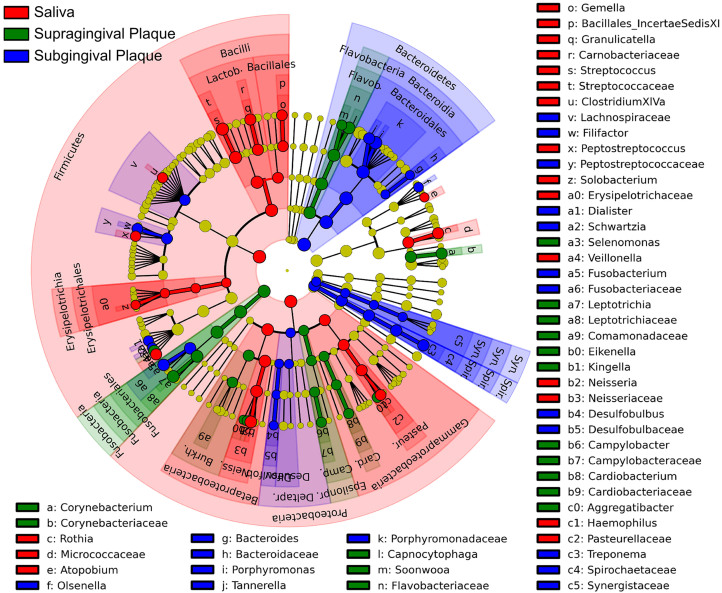
Habitat-specific taxa detected by LEfSe. Color indicates the oral habitat in which each differential clade was most abundant.

To identify the distinguishing taxa in human saliva, supragingival and subgingival plaque, Mann-Whitney U test and LEfSe were performed based on the RDP taxonomy data. In saliva, the most abundant phylum was *Firmicutes*, followed by *Bacteroidetes* and *Proteobacteria*. However, in both supra and subgingival plaque, the top three phyla in abundance were *Bacteroidetes*, *Firmicutes* and *Fusobacteria* (see Supplementary Table S3 online). At the genus level, as shown by a circular cladogram constructed by LEfSe, *Steptococcus*, *Neisseria*, *Veillonella*, *Rothia*, *Granulicatella*, *Haemophilus* and *Gemella* were significantly enriched in the microbiota of saliva. Several facultative anaerobic genera, including *Capnocytophaga*, *Leptotrichia* and *Corynebacterium*, were predominant in supragingival plaque. Obligate anaerobic genera including *Porphyromonas*, *Treponema*, *Tannerella*, *Fusobacterium* and *Filifactor* were more abundant in subgingival plaque (Figure 2, Supplementary Table S3).

### Key species-level phylotypes associated with periodontitis and two clinical parameters

**Figure 3:**
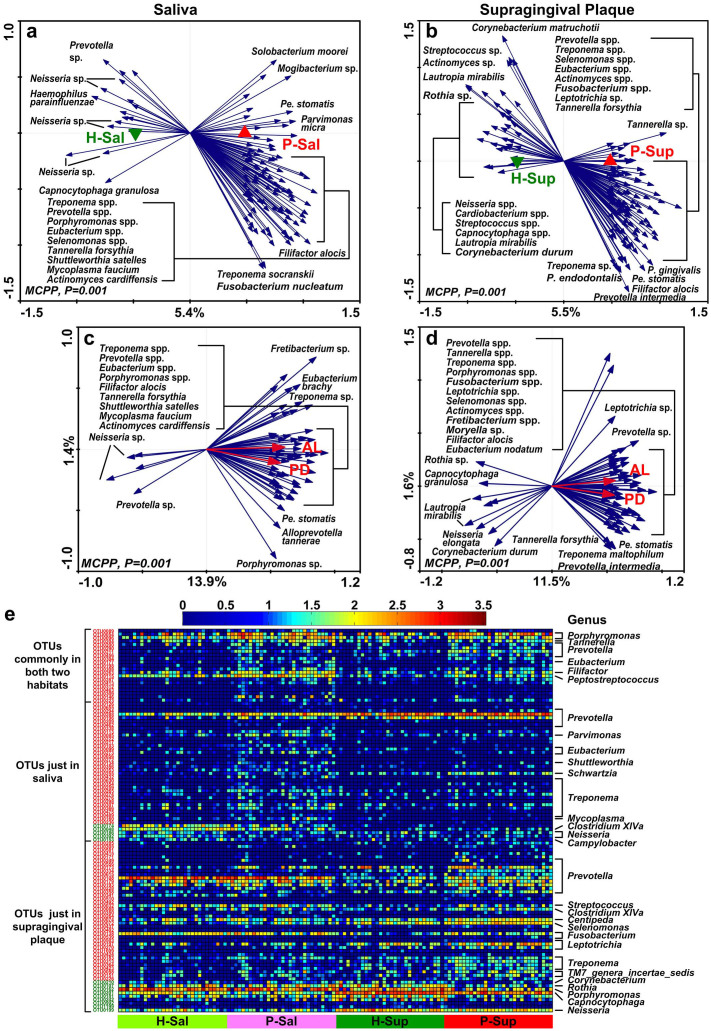
Key phylotypes associated with periodontitis identified by two-step RDA. Bioplot of RDA of the microbiota in saliva (a) and supragingival plaque (b) relative to periodontitis (first step of RDA). Responding OTUs that have at least 6% of the variability of the oral microbiota are indicated by blue arrows. (c–d) Bioplot of RDA of the periodontitis-associated OTUs identified in (a) and (b) relative to the combination of pocket depth (PD) and attachment loss (AL) (second step of RDA). Responding OTUs that have at least 11% and 9% of the variability of the oral microbiota in saliva and supragingival plaque respectively, are indicated by blue arrows. (e) Relative abundances of the 110 key OTUs identified by two steps of RDA (c and d). OTUs shown in green are more abundant in healthy volunteers and those in red are more abundant in periodontitis. The color of the spot corresponds to the normalized and log-transformed relative abundance of the OTU. The genus names of the OTUs are shown on the right. P, *Porphyromonas*; Pe, *Peptostreptococcus*.

To identify the species-level phylotypes of the oral microbiota associated with periodontitis, two-step redundancy analysis (RDA) was performed for saliva and supragingival plaque samples from periodontitis and healthy individuals. At the first step of RDA, health and periodontitis were used as environmental variables and the relative abundances of all OTUs in saliva or supragingival plaque were used as species variables. The Monte Carlo Permutation Procedure (MCPP) showed that the difference of microbiota between periodontitis and health in saliva or supragingival plaque was significant (*P* = 0.001) (Figure 3a, b). We identified 123 and 148 OTUs explaining more than 6% of the variability of the oral microbiota as key phylotypes associated with periodontitis in saliva and supragingival plaque, respectively. In the second step of RDA, two clinical parameters, pocket depth and attachment loss, were used as environment variables, and the relative abundances of OTUs selected in the first step of RDA were used as species variables (Figure 3c, d). We identified 61 and 70 OTUs as key phylotypes closely relevant to the clinical parameters in saliva and supragingival plaque, respectively. OTUs identified from those two habitats were merged into 110 OTUs all together.

Notably, 21 of the above 110 OTUs were identified in both saliva and supragingival plaque, and all 21 were enriched in periodontitis (Figure 3e, Supplementary Table S4 and S5). Eighteen of the 21 OTUs had nearest neighbors with similarity over 99% in the Human Oral Microbiome Database (HOMD), including *Porphyromonas gingivalis*, *Porphyromonas endodontalis*, *Tannerella forsythia*, *Prevotella* sp., *Eubacterium nodatum*, *Filifactor alocis*, and *Peptostreptococcus stomatis*. The other three OTUs had lower similarities (<97%) with existing hit taxa in the database, suggesting that they are likely novel phylotypes (see Supplementary Table S4 online). Though the lone-pair subgingival plaque samples of periodontitis patients were not involved in RDA, it was found that those 21 OTUs also represented the major subgingival microbiome of periodontitis, accounting for 31.80% of the microbial community in P-Sub. Moreover, 14 of the 21 OTUs were present in two-thirds of subgingival plaque samples (see Supplementary Table S6 online), and OTU0054, an identified phylotype showing 100% similarity to *P. gingivalis*, accounted for 18.70% of the microbial community in P-Sub, constituting the most abundant phylotype in subgingival plaque.

### Co-occurrence pattern of key periodontitis-associated phylotypes in the oral microbiota

**Figure 4:**
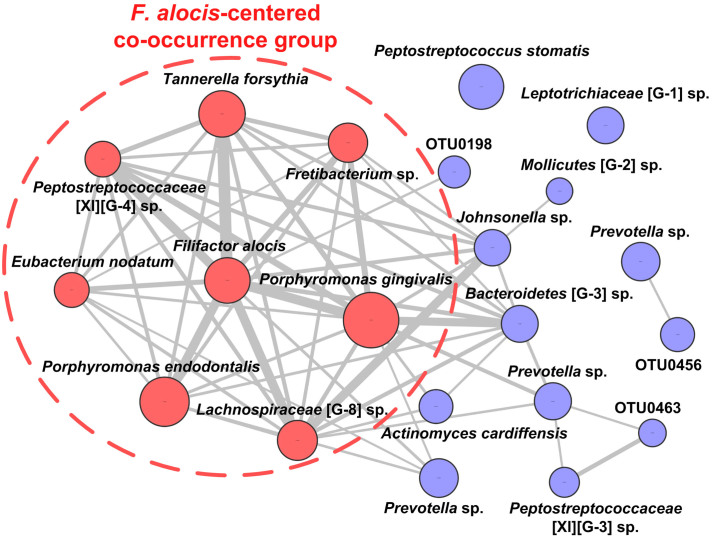
Co-occurrence network of 21 key OTUs in the oral microbiota. Nodes represent the OTUs identified by two-step RDA, and their OTU number or nearest neighbors are displayed around the nodes. The size of the node corresponds to the log-transformed relative abundance of the OTU. Each pair of OTUs showing a Spearman's correlation coefficient value not lower than 0.5 is linked with a connecting line whose thickness corresponds to the coefficient values. Eight OTUs displaying high correlation coefficients with each other (R ≥ 0.5) are colored with red (*F. alocis*-centered co-occurrence group), others are blue.

Based on the 21 periodontitis-relevant species, we investigated the correlations of each pair of them. Spearman's correlation coefficients were assessed based on the normalized relative abundances of the 21 periodontitis-associated phylotypes using all 150 oral samples from the three distinct oral niches of the two cohorts. Interestingly, eight OTUs displayed high correlation coefficients with each other (R ≥ 0.5, *P* < 0.05). Notably, strong positive correlation (R > 0.6, *P* < 0.05) was observed between OTU0263 and the remaining seven OTUs (Figure 4, Supplementary Fig. S3). Thus, OTU0263, with 100% similarity to *Filifactor alocis*, together with seven OTUs (OTU0054, OTU0096, OTU0204, OTU0177, OTU0223, OTU0308 and OTU0413) which were closely related to *P. gingivalis*, *P. endodontalis*, *T. forsythia*, *E. nodatum*, *Fretibacterium* sp., *Lachnospiraceae* [G-8] sp. and *Peptostreptococcaceae* [XI][G-4] sp., seemed to form a *F. alocis*-centered bacterial cluster with co-occurrence relationships in the human oral cavity. This *F. alocis*-centered co-occurrence group collectively represented 6.28%, 5.49% and 29.11% of the oral microbiota in P-Sal, P-Sup and P-Sub, respectively.

### Diagnostic value assessment of the *F. alocis*-centered co-occurrence group

**Figure 5:**
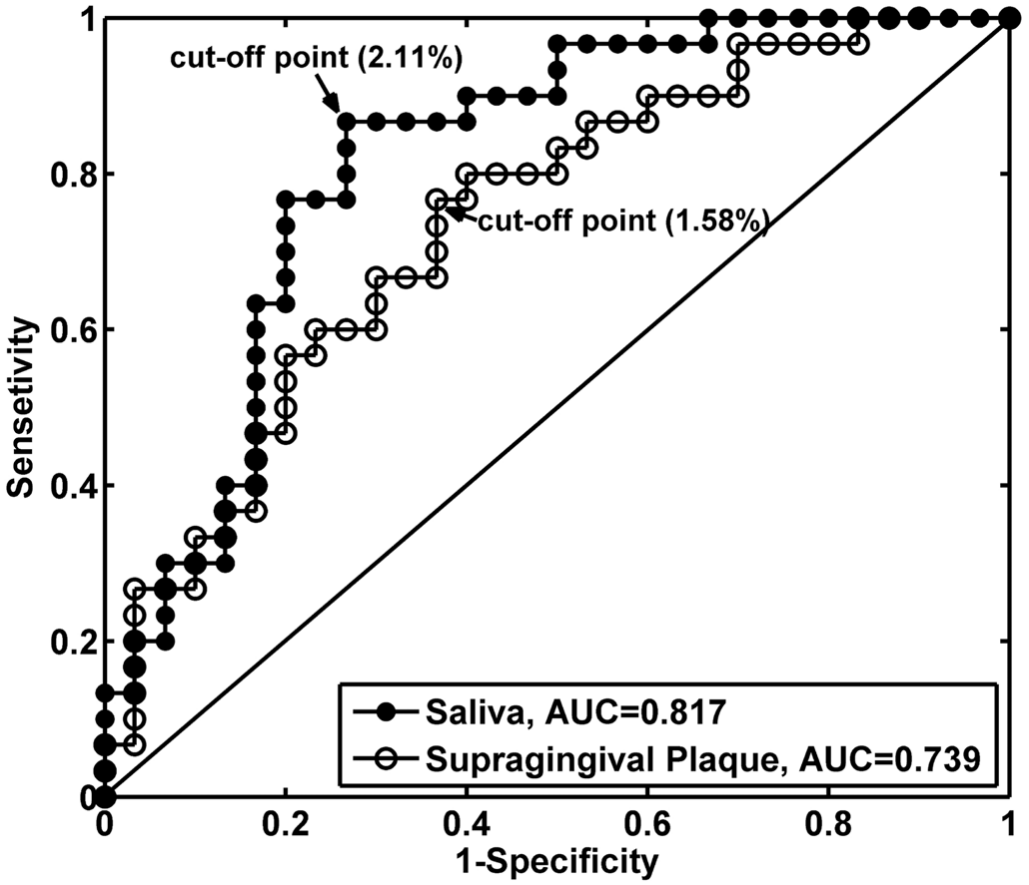
ROC curves of the *F. alocis*-centered co-occurrence group in the identification of periodontitis based on its relative abundance in saliva or supragingival plaque. The areas under the ROC curve (AUC) of the co-occurrence group in saliva and supragingival plaque were calculated and marked in the lower right corner. The arrows point out the optimal cut-off point in the microbiota of saliva or supragingival plaque.

To assess its performance in identifying the risk of periodontitis, the relative abundances of the *F. alocis*-centered co-occurrence group and the 21 individual key phylotypes were used to construct receiver operating characteristic (ROC) curves in which test sensitivity is plotted against 1-specificity or false positive rate (FPR), to evaluate the performance of diagnostic tests. Area under the ROC curve, cut-off point, sensitivity, specificity, likelihood ratio and P values were calculated and compared. In saliva, the area under the ROC curve of the co-occurrence group was the highest of all the tested phylotypes (0.817), implying that this cluster has the highest value for periodontitis diagnosis (Figure 5, Supplementary Table S7). The cut-off points of the abundances of this group were determined at 2.11% in saliva (Chi-square test, *P* < 0.001, sensitivity 0.87, specificity 0.73, likelihood 23.8) and 1.58% in supragingival plaque (Chi-square test, *P* < 0.001, sensitivity 0.77, specificity 0.63, likelihood 10.1), indicating that a person who has the abundance of this cluster over 2.11% in saliva or over 1.58% in supragingival plaque would have a high risk of periodontitis.

## Discussion

For decades, efforts have been made to identify potential pathogens and elucidate their functions in the development of periodontitis. However, given the complexity of the oral microbiota, there is still no definitive evidence demonstrating how many different pathogens are involved in the polymicrobial disease and how these co-infecting species interact with each other in triggering the disease and contributing to its progression.

In this investigation, saliva, supragingival plaque and subgingival plaque samples were used to characterize the oral microbiota. *Streptococcus*, *Leptotrichia* and *Actinomyces* were commonly detected in all 150 samples with similar abundance in health and periodontitis. Such taxa are regarded as components of the core oral microbiota that adapt to the oral environment and provide structural support for the oral microbiota^11^. These three genera have been identified as the core members or major genera with the largest representation in the oral microbiome in previous studies^2^,^24^,^25^.

We also found that each oral habitat harbored its distinct microbiome. In saliva, several genera including *Steptococcus*, *Neisseria*, *Veillonella*, *Rothia*, *Granulicatella*, *Haemophilus* and *Gemella* were predominant, which were previously reported as commensal bacteria colonizing the mucosal surfaces of mouth^17^,^26^. Supragingival plaque exhibited the highest level of microbial diversity, dominated by three facultative anaerobic genera including *Capnocytophaga*, *Leptotrichia* and *Corynebacterium*. In subgingival plaque, obligate anaerobic bacteria such as *Porphyromonas*, *Treponema*, *Tannerella*, *Fusobacterium* and *Filifactor* were found as major members of this anaerobic niche. Thus, the different microbial distribution in diverse oral habitats may be mainly driven by the surface structure and oxygen condition of each microhabitat^27^.

Via a microbiome-wide association study (MiWAS) strategy, 21 OTUs were found to be associated with periodontitis. MiWAS strategy combines high-throughput DNA-sequencing technology with multivariate statistical tools such as principal component analysis (PCA), principal coordinate analysis (PCoA) and redundancy analysis (RDA), to explore the structural variations across the entire human microbiome without any prior hypothesis, aiming to identify all relevant bacteria associated with diseases^28^,^29^. Here, using pyrosequencing data from the bacterial 16S rRNA gene V3 region, 21 periodontitis-enriched OTUs were detected through a two-step RDA in both saliva and supragingival plaque. In subgingival plaque, these 21 OTUs collectively represented about one-third of the microbiota and contained the most abundant phylotype (OTU0054) of this habitat. Hence, these 21 OTUs were closely linked to periodontitis and could be considered as potential pathogens.

Despite the difficulty of assigning an OTU to an exact species due to the short sequencing read lengths, OTU0054, OTU0204 and OTU0263 were found to be closely related to the putative periodontopathic bacteria *P. gingivalis*, *T. forsythia* and *F. alocis*, and were present in 93.3%, 90.0% and 92.2% of oral samples from periodontitis patients, respectively. These three OTUs accounted for 65.7% of the total abundance of the 21 OTUs in the three habitats of periodontitis patients. *P. gingivalis*, *T. forsythia* and *F. alocis* were frequently found to link with periodontitis in previous studies using subgingival plaque samples^7^,^9^,^10^,^11^,^30^ or saliva samples^31^,^32^. Among those three bacteria, *P. gingivalis* was the most widely studied periodontal pathogen, partially due to its high detection rate via culture methods^4^,^5^. This bacterium shows numerous virulence factor activities, such as gingipain proteases, lipopolysaccharides and fimbriae^33^, allowing it to invade and survive within gingival epithelial cells. It was reported that even at low colonization levels (< 0.01% of the total microbiota), *P. gingivalis* could change the amount and the composition of the oral microbial symbionts, triggering inflammatory periodontal bone loss in a mouse model^34^.

In addition, we identified 18 other OTUs significantly associated with this disease, including OTUs that were closely related to *P. endodontalis* (OTU0096), *E. nodatum* (OTU0177), *Peptostreptococcus* spp. (OTU0115, OTU0413 and OTU0312), *Prevotella* spp. (OTU0200, OTU0290 and OTU0211), *Lachnospiraceae* [G-8] sp. (OTU0308), *Bacteroidetes* [G-3] sp. (OTU0304), *Fretibacterium* sp. (OTU0223), *Johnsonella* sp. (OTU0287), *Leptotrichiaceae* [G-1] sp. (OTU0146) and *Mollicutes* [G-2] sp. (OTU0309). These 14 phylotypes were previously reported to be associated with periodontitis in investigations based only on subgingival plaque samples^9^,^10^,^11^, while they were detected in all three oral habitats in this study. We also observed that one OTU (OTU0089), which increased its abundance in periodontitis, is closely related to *Actinomyces cardiffensis*, a Gram-positive facultative anaerobic rod. This bacterium can be isolated from diverse human clinical sources such as pleural fluid, brain, jaw, pericolic and ear abscesses^35^, and was reported to be involved in human infection cases such as pulmonary and hepatic actinomycosis^36^,^37^, but its linkage with periodontitis has not previously been reported in the literature. We suspected that its capability of forming abscesses in diseased lesions might contribute to the invasion of itself or other pathogens into the periodontal tissues, promoting the progression of periodontitis. Finally, we found three OTUs (OTU0456, OTU0198 and OTU0463) with no known nearest neighbor that were significantly enriched in periodontitis. These three newly identified phylotypes represent 1.27% of the total abundance of the 21 OTUs in all samples of patients. Our results reinforce the polymicrobial nature of periodontitis as a disease related to a dysbiotic microbiota, with dozens of microbes implicated.

For a polymicrobial disease, identification of potential pathogens is only the first step; delineating the species-species interactions of multiple pathogens is essential for understanding the microbial etiology of the disease. In the present study, we examined the pairwise correlation coefficient among the 21 potential periodontal pathogens identified, according to their abundance in all 150 oral samples from three distinct oral niches of the two human cohorts. A bacterial group composed of eight phylotypes showed a robust co-occurrence pattern. Their abundance varied among different oral samples with the same tendency, increasing together in three habitats of periodontitis patients and decreasing together in two habitats of healthy individuals. Our finding implied there might be synergistic interactions among them, which should be confirmed by further *in vitro* or animal experiments.

The synergistic interactions between some cultivable indigenous oral pathogens have been revealed previously by *in vitro* studies. Grenier demonstrated that *P. gingivalis* can produce isobutyric acid to stimulate the growth of *T. denticola* and metabolize the succinate produced by *T. denticola* to promote its own growth^38^. *F. nucleatum* can maintain a more neutral pH in the microenvironment to protect acid-sensitive species such as *P. gingivalis* against acid attack^39^ or generate a necessary reductive and capnophilic microenvironment for the growth of *P. gingivalis*^40^. The ability of *P. gingivalis* to invade human gingival epithelial cell was enhanced when co-cultured with *F. nucleatum*^41^. However, these studies focused only on interactions between two arbitrarily selected potential pathogens. It is not clear whether these selected pathogens actually co-occur in the same niche in disease. The co-occurrence pattern found in our study indicates that it is necessary to look at interactions among pathogens that occur in the same habitat to evaluate relevance to disease progression. It is also necessary to study interactions among more than two potential pathogens.

In the co-occurrence pattern we identified, a phylotype closely related to *F. alocis* showed strong positive correlations with seven other members (R > 0.6 for each pair), thus seemed to be the center of this bacteria cluster. *F. alocis* is a Gram-positive obligate anaerobic rod, which was recently identified as one of the marker organisms for periodontal diseases using culture-independent approaches, due to its higher prevalence and abundance in periodontal disease sites compared with healthy sites^7^,^10^,^42^,^43^. This organism was even found to rank higher in its prevalence than previously identified periodontal pathogens, including *P. gingivalis*^44^. *F. alocis* is relatively resistant to oxidative stress and possesses several virulence factors, such as sialoglycoproteases and proteases, which may partially contribute to its colonization and survival in the periodontal pocket. In addition, *in vitro* experiments showed that *F. alocis* could induce apoptosis in gingival epithelial cells (GECs) and stimulate pro-inflammatory cytokines such as IL-1β, IL-6 and TNF-α secreted by GECs^45^, supporting the notion that *F. alocis* may be an important pathogen in the formation of subgingival biofilm and the onset of periodontal diseases. Previous studies also showed that *F. alocis* could synergistically interact with other periodontal pathogens *in vitro*. For example, *F. alocis* could enhance the biofilm formation and the invasion of epithelial cells by *P. gingivalis* during coculture^44^. *F. alocis* was also found accumulated around *F. nucleatum*-abundant regions and showed mutualistic growth with *F. nucleatum* in an *in vitro* community development model^46^. Given the co-occurrence pattern we found, *F. alocis* may similarly enhance the growth or virulence of other pathogens in the co-occurrence group, promoting the development of periodontitis.

The remaining seven members of the co-occurrence group include *P. gingivalis*, *P. endodontalis*, *T. forsythia*, *E. nodatum*, *Fretibacterium* sp., *Lachnospiraceae* [G-8] sp. and *Peptostreptococcaceae* [XI][G-4] sp. Each of these bacteria has been previously identified as associated with periodontitis in different studies by culture or culture-independent approaches^5^,^9^,^11^,^47^,^48^. Though they are not novel pathogens for periodontitis, their coordinated occurrence is novel. *P. gingivalis* and *T. forsythia* were the only two microorganisms which were reported to be synergistic in previous studies. For example, *in vitro* experiment showed that *P. gingivalis* or its outer membrane vesicles could enhance the attachment and invasion of *T. forsythia* to epithelial cells^49^. Yoneda *et al.* also reported that the co-infection of *P. gingivalis* and *T. forsythia* induced greater abscess size than did mono-infection of each bacterium in a murine model^50^. There are no relevant reports about interactions among the remaining members of the co-occurrence group. It is possible that some of them produce metabolites that directly affect the growth of others (metabolite cross-feeding) or that modify the local microenvironment to favor the growth or virulence of other organisms^51^. The inherent relationship between these multiple potential pathogens in the oral microbiota merits further study.

The *F. alocis*-centered co-occurrence group potentially has a high diagnostic value for periodontitis, especially in saliva where the cluster showed the largest area under the ROC curve and high sensitivity and specificity to discriminate periodontitis from healthy samples (cut-off point 2.11%, *P* < 0.001, sensitivity 0.87, specificity 0.73, likelihood 23.8). Saliva is an abundant oral biofluid that can be collected noninvasively. Previous reports have revealed that oral bacteria such as *A. actinomycetemcomitans*, *C. rectus*, *P. gingivalis*, *P. intermedia*, *P. nigrescens*, *P. micros*, *T. denticola*, *T. forsythia*, *F. nucleatum* and *T. socranskii* are detectable and enriched in saliva from periodontitis patients, and therefore could be useful salivary biomarkers of periodontitis^31^,^52^. We demonstrate in this study that the diagnostic value of the *F. alocis*-centered co-occurrence group was higher than any single periodontitis-related phylotype in saliva, reinforcing the significant role of this group in periodontitis progression and providing a potential indicator to identify high-risk periodontitis patients.

In summary, we characterized the microbiota of three main oral habitats in human subjects with and without periodontitis. Saliva, supragingival plaque and subgingival plaque harbor *Streptococcus*, *Leptotrichia* and *Actinomyces* as a common core oral microbiota, and each habitat also harbors its distinct microbial members. Twenty-one species-level OTUs were identified as periodontitis-associated phylotypes, including a *F. alocis*-centered co-occurrence group consisting of eight phylotypes that were positively correlated with each other in various habitats of the oral cavity. The co-occurrence pattern of those potential pathogens underlines their ecological relationship relevant to disease progression. The findings from this study may serve as an ecological framework to facilitate not only the understanding of the complex etiology of periodontitis, but also the development of a new periodontal diagnoses and therapies targeting this co-occurrence group.

## Methods

### Study subjects

A total of 30 periodontitis patients, aged 29–67 years, were recruited from the department of periodontology in Ninth People's Hospital (Shanghai, China). These patients took no periodontal treatment in the 3 months before sample collection. Thirty periodontally healthy volunteers, aged 21–55 years, were selected as controls. Inclusion criteria for all participants included no pregnancy or any systemic diseases, no antibiotic therapy within the past 3 months, and at least 20 teeth in the oral cavity excluding third molars. Every periodontitis patient had, in each quadrant, at least one posterior tooth with pocket depth ≥4 mm and attachment loss ≥2 mm, whereas healthy controls exhibited no site with pocket depth >3 mm or attachment loss ≥2 mm and displayed no clinical gingival inflammation (no more than 10% of the sites with bleeding on probing and absence of gingival redness/edema). The baseline clinical characteristics of all individuals are showed in Table 1. Written informed consent was obtained from all the participants. This study was approved by the Ethics Committee of Shanghai Ninth People's Hospital affiliated to Shanghai Jiao Tong University, School of Medicine, China (Document No. 201262), and all experiments were performed in accordance with the relevant guidelines and regulations.

### Sample collection

All the participants were instructed to refrain from eating, drinking and any oral hygiene practice for at least 3 h before sampling. After rinsing mouth with water, approximately 1 ml non-stimulated saliva was collected and transferred into 1.5 ml sterile centrifuge tubes. The selected teeth (four teeth with the highest level of pocket depth from four quadrants) were then isolated using cotton rolls and gentle air-drying. Supragingival plaque was collected first, followed by subgingival plaque from the bottom of the periodontal pocket of the same teeth using sterile subgingival curettes. The pooled supragingival plaque and the pooled subgingival plaque from each subject were separately stored in 1.5 ml sterile centrifuge tubes containing 1 ml sterile phosphate-buffered saline (PBS). All samples were immediately frozen at −80°C until DNA extraction. In total, 150 samples were collected and divided into five groups according to the sampling habitats and populations: H-Sal (saliva samples collected from healthy controls, n = 30), H-Sup (supragingival plaque samples collected from healthy controls, n = 30), P-Sal (saliva samples collected from periodontitis patients, n = 30), P-Sup (supragingival plaque samples collected from periodontitis patients, n = 30), P-Sub (subgingival plaque samples collected from periodontitis patients, n = 30). Subgingival plaque samples were not collected from healthy controls because there is no distinct periodontal pocket formed in healthy gingival tissue.

### DNA extraction and pyrosequencing

Oral samples were washed twice with PBS and underwent two cycles of freeze-thawing at −80°C for 10 min and 60°C for 2 min. Genomic DNA was then extracted from each oral sample using bead-beating extraction and phenol-chloroform purification, as described previously^28^,^53^. The extracted DNA was used as a template for PCR amplification. The bacterial universal primer pair^54^ consisting of the forward primer 5′-NNNNNNNNCCTACGGGAGGCAGCAG-3′ and the reverse primer 5′-NNNNNNNNATTACCGCGGCTGCT-3′, marked at the 5′ end with a sample-unique 8-base barcode, was used to amplify the V3 region of 16S ribosomal RNA gene from each oral sample. PCR amplification, pyrosequencing of the PCR amplicons, and quality control of raw data were performed as described previously^55^. Briefly, we performed the following steps to select the high quality reads for bioinformatics analysis: 1) Search for the primers by using blast-based matching (Word size = 4, E-value = 0.1); the primer at least at the sequencing end should exist. 2) Locate the barcodes according to the position of the primers; reads should have at least one complete barcode; reads with incomplete barcodes at both ends, or with complete but poorly-matched barcode pairs (more than one insertion/deletion/mismatch) were discarded. 3) Reads were assigned to the corresponding sample using the complete barcode. If the barcodes were complete at both ends but mismatched with each other, we took the barcode at the sequencing end. 4) After trimming the primer and barcode bases, those sequences with a variable region more than 100nt and less than 300nt in length and with no more than two undetermined bases were preserved.

### Bioinformatics and multivariate statistics

All high-quality pyrosequencing reads were aligned against the Greengenes database using the nearest alignment space termination (NAST) algorithm^56^ with template length ≥ 90 bases and percent identity ≥ 75%. After that, the remaining sequences were clustered using CD-hit with 99.9% similarity^57^. The most abundant sequence of each cluster was selected as the representative sequence and subjected to online RDP classifier (Version 2.6) for taxonomical assignment with a bootstrap cutoff of 50% (http://rdp.cme.msu.edu/classifier/classifier.jsp). The data of RDP taxonomy was used in LEfSe to discover the taxa differentiating the microbial communities specific to different oral habitats^58^. Briefly, saliva, supragingival plaque and subgingival plaque were used as three classes and divided into two subclasses according to the sampling populations (periodontitis/health). After factorial Kruskal-Wallis test among classes and subsequent pairwise Wilcoxon test between subclasses at the same population with a significance alpha of 0.01 and an effect size threshold of 2, the taxa, which showed highest level of abundances in one oral habitat, were selected.

Meanwhile, those representative sequences after CD-hit were imported into the ARB to construct a neighbor-joining tree. Operational taxonomic unit (OTU) was delineated at 96% similarity level with DOTUR^59^. Rarefaction analysis (aRarefactWin software, S Holland, Stratigraphy Laboratory, University of Georgia, Athens, GA, USA, http://www.uga.edu/strata/software), Shannon diversity index (H') (R package 2.12.0), as well as Simpson's diversity index (1-D) and Chao1 estimate were calculated (QIIME). The most abundant sequence of each OTU was selected, imported into the ARB and inserted into a pre-established phylogenetic tree of the full-length 16S rRNA gene sequences to generate a phylogenetic tree. The phylogenetic tree, together with sequence abundance data, was then used for online Fast UniFrac analysis based on a weighted metric (http://unifrac.colorado.edu/). The most abundant sequence of each OTU was BLAST searched against the Human Oral Microbiome Database (HOMD) to find out the nearest neighbor of the OTU (http://www.homd.org/modules.php?op=modload&name=RNAblast&file=index&link=upload). Relative abundances of OTUs were used for correlation analysis via Matlab R2010a (The MathWorks, Inc., USA). Redundancy analysis (RDA) was performed using CANOCO for Windows 4.5 (Microcomputer Power, USA). ROC curves were constructed to determine the diagnostic values for periodontitis of key periodontitis-associated phylotypes or co-occurrence group via SPSS Statistics 21 (IBM, USA). The area under the ROC curve is the most commonly used index to evaluate the diagnostic accuracy, with values close to 1.0 indicating high diagnostic accuracy. The points displaying the largest Youden's index (sensitivity + specificity - 1) were defined as the optimal cut-off points.

## Author Contributions

Conceived and designed the experiments: X.P., Z.T. and L.Z. Performed the experiments: H.C. Analyzed the data: H.C. Contributed reagents/materials/analysis tools: Z.T., Y.L., Z.Q., M.Z. and G.W. Wrote the paper: H.C. and X.P. Revised the manuscript: X.P., L.Z. and L.B.

## Additional information

These sequence data have been uploaded to the Sequence Read Archive database under the accession number SRP050141.

## Supplementary Material

Supplementary Information Supplementary information

## Figures and Tables

**Table 1:** Background characteristics of all individuals

	Periodontitis patients	Periodontally healthy volunteers
**N**	30	30
**Gender (Males/Females)**	15/15	9/21
**Age, years**	29–67	21–55
**Pocket depth (mean ± SD, mm)**	4.80 ± 0.96	2.13 ± 0.41
**Attachment loss (mean ± SD, mm)**	4.30 ± 1.43	0.10 ± 0.31
**Sampling habitats**	Saliva, Supragingival plaque, Subgingival plaque	Saliva, Supragingival plaque

## References

[b1] Pihlstrom B. L., Michalowicz B. S. & Johnson N. W. Periodontal diseases. Lancet 366, 1809–1820 (2005). 16298220 10.1016/S0140-6736(05)67728-8

[b2] Zarco M. F., Vess T. J. & Ginsburg G. S. The oral microbiome in health and disease and the potential impact on personalized dental medicine. Oral Dis. 18, 109–120 (2012). 21902769 10.1111/j.1601-0825.2011.01851.x

[b3] Teles R., Teles F., Frias-Lopez J., Paster B. & Haffajee A. Lessons learned and unlearned in periodontal microbiology. Periodontol. 2000 62, 95–162 (2013). 23574465 10.1111/prd.12010 PMC3912758

[b4] Dzink J. L., Socransky S. S. & Haffajee A. D. The predominant cultivable microbiota of active and inactive lesions of destructive periodontal diseases. J. Clin. Periodontol. 15, 316–323 (1988). 3292595 10.1111/j.1600-051x.1988.tb01590.x

[b5] Moore W. E. C. & Moore L. V. H. The bacteria of periodontal diseases. Periodontol. 2000 5, 66–77 (1994). 9673163 10.1111/j.1600-0757.1994.tb00019.x

[b6] Socransky S. S., Haffajee A. D., Cugini M. A., Smith C. & Kent R. L. Microbial complexes in subgingival plaque. J. Clin. Periodontol. 25, 134–144 (1998). 9495612 10.1111/j.1600-051x.1998.tb02419.x

[b7] Kumar P. S., Griffen A. L., Moeschberger M. L. & Leys E. J. Identification of candidate periodontal pathogens and beneficial species by quantitative 16S clonal analysis. J. Clin. Microbiol. 43, 3944–3955 (2005). 16081935 10.1128/JCM.43.8.3944-3955.2005 PMC1233920

[b8] Ledder R. G. *et al.* Molecular analysis of the subgingival microbiota in health and disease. Appl. Environ. Microbiol. 73, 516–23 (2007). 17085691 10.1128/AEM.01419-06 PMC1796972

[b9] Colombo A. P. *et al.* Comparisons of subgingival microbial profiles of refractory periodontitis, severe periodontitis, and periodontal health using the human oral microbe identification microarray. J. Periodontol. 80, 1421–32 (2009). 19722792 10.1902/jop.2009.090185 PMC3627366

[b10] Griffen A. L. *et al.* Distinct and complex bacterial profiles in human periodontitis and health revealed by 16S pyrosequencing. ISME J. 6, 1176–1185 (2012). 22170420 10.1038/ismej.2011.191 PMC3358035

[b11] Abusleme L. *et al.* The subgingival microbiome in health and periodontitis and its relationship with community biomass and inflammation. ISME J. 7, 1016–1025 (2013). 23303375 10.1038/ismej.2012.174 PMC3635234

[b12] Ge X., Rodriguez R., Trinh M., Gunsolley J. & Xu P. Oral microbiome of deep and shallow dental pockets in chronic periodontitis. PLoS One 8, e65520 (2013). 23762384 10.1371/journal.pone.0065520 PMC3675156

[b13] Wang J. *et al.* Metagenomic sequencing reveals microbiota and its functional potential associated with periodontal disease. Sci. Rep. 3, 1843 (2013). 23673380 10.1038/srep01843 PMC3654486

[b14] Li Y. *et al.* Phylogenetic and functional gene structure shifts of the oral microbiomes in periodontitis patients. ISME J. 8, 1879–1891 (2014). 24671083 10.1038/ismej.2014.28 PMC4139721

[b15] Duran-Pinedo A. E. *et al.* Community-wide transcriptome of the oral microbiome in subjects with and without periodontitis. ISME J. 8, 1659–1672 (2014). 24599074 10.1038/ismej.2014.23 PMC4817619

[b16] Parahitiyawa N. B. *et al.* Exploring the oral bacterial flora: current status and future directions. Oral Dis. 16, 136–145 (2010). 19627515 10.1111/j.1601-0825.2009.01607.x

[b17] Mager D. L., Ximenez-Fyvie L. A., Haffajee A. D. & Socransky S. S. Distribution of selected bacterial species on intraoral surfaces. J. Clin. Periodontol. 30, 644–654 (2003). 12834503 10.1034/j.1600-051x.2003.00376.x

[b18] Avila M., Ojcius D. M. & Yilmaz O. Z. The oral microbiota: living with a permanent guest. DNA Cell Biol. 28, 405–411 (2009). 19485767 10.1089/dna.2009.0874 PMC2768665

[b19] Filoche S., Wong L. & Sissons C. H. Oral biofilms: emerging concepts in microbial ecology. J. Dent. Res. 89, 8–18 (2010). 19918089 10.1177/0022034509351812

[b20] Short F. L., Murdoch S. L. & Ryan R. P. Polybacterial human disease: the ills of social networking. Trends Microbiol. 22, 508–516 (2014). 24938173 10.1016/j.tim.2014.05.007 PMC4158425

[b21] Haffajee A. D. & Socransky S. S. Microbial etiological agents of destructive periodontal diseases. Periodontol. 2000 5, 78–111 (1994). 9673164 10.1111/j.1600-0757.1994.tb00020.x

[b22] Kesavalu L. *et al.* Rat model of polymicrobial infection, immunity, and alveolar bone resorption in periodontal disease. Infect. Immun. 75, 1704–1712 (2007). 17210663 10.1128/IAI.00733-06 PMC1865722

[b23] Zhu Y. *et al.* *Porphyromonas gingivalis* and *Treponema denticola* synergistic polymicrobial biofilm development. PLoS One 8, e71727 (2013). 23990979 10.1371/journal.pone.0071727 PMC3753311

[b24] Zaura E., Keijser B. J. F., Huse S. M. & Crielaard W. Defining the healthy “core microbiome” of oral microbial communities. BMC Microbiol. 9, 259 (2009). 20003481 10.1186/1471-2180-9-259 PMC2805672

[b25] Bik E. M. *et al.* Bacterial diversity in the oral cavity of 10 healthy individuals. ISME J. 4, 962–974 (2010). 20336157 10.1038/ismej.2010.30 PMC2941673

[b26] Keijser B. J. F. *et al.* Pyrosequencing analysis of the oral microflora of healthy adults. J. Dent. Res. 87, 1016–1020 (2008). 18946007 10.1177/154405910808701104

[b27] Segata N. *et al.* Composition of the adult digestive tract bacterial microbiome based on seven mouth surfaces, tonsils, throat and stool samples. Genome Biol. 13, R42 (2012). 22698087 10.1186/gb-2012-13-6-r42 PMC3446314

[b28] Wang T. *et al.* Structural segregation of gut microbiota between colorectal cancer patients and healthy volunteers. ISME J. 6, 320–329 (2012). 21850056 10.1038/ismej.2011.109 PMC3260502

[b29] Zhao L. The gut microbiota and obesity: from correlation to causality. Nat. Rev. Microbiol. 11, 639–647 (2013). 23912213 10.1038/nrmicro3089

[b30] Paster B. J. *et al.* Bacterial diversity in human subgingival plaque. J. Bacteriol. 183, 3770–3783 (2001). 11371542 10.1128/JB.183.12.3770-3783.2001 PMC95255

[b31] Brinkmann O., Zhang L., Giannobile W. V. & Wong D. T. Salivary biomarkers for periodontal disease diagnostics. Expert Opin. Med. Diagn. 5, 25–35 (2011). 23484474 10.1517/17530059.2011.542144

[b32] Belstrøm D. *et al.* Differences in bacterial saliva profile between periodontitis patients and a control cohort. J. Clin. Periodontol. 41, 104–112 (2014). 24303924 10.1111/jcpe.12190

[b33] Holt S. C., Kesavalu L., Walker S. & Genco C. A. Virulence factors of *Porphyromonas gingivalis*. Periodontol. 2000 20, 168–238 (1999). 10522227 10.1111/j.1600-0757.1999.tb00162.x

[b34] Hajishengallis G. *et al.* Low-abundance biofilm species orchestrates inflammatory periodontal disease through the commensal microbiota and complement. Cell Host Microbe 10, 497–506 (2011). 22036469 10.1016/j.chom.2011.10.006 PMC3221781

[b35] Hall V., Collins M. D., Hutson R., Falsen E. & Duerden B. I. *Actinomyces cardiffensis* sp. nov. from human clinical sources. J. Clin. Microbiol. 40, 3427–3431 (2002). 12202588 10.1128/JCM.40.9.3427-3431.2002 PMC130680

[b36] Seo J. Y., Yeom J.-S. & Ko K. S. *Actinomyces cardiffensis* septicemia: a case report. Diagn. Microbiol. Infect. Dis. 73, 86–88 (2012). 22445645 10.1016/j.diagmicrobio.2012.02.012

[b37] Wakabayashi K. *et al.* Pulmonary actinomycosis caused by *Actinomyces cardiffensis*. Intern. Med. 51, 2929–2931 (2012). 23064570 10.2169/internalmedicine.51.7997

[b38] Grenier D. Nutritional interactions between two suspected periodontopathogens, *Treponema denticola* and *Porphyromonas gingivalis*. Infect. Immun. 60, 5298–5301 (1992). 1333450 10.1128/iai.60.12.5298-5301.1992 PMC258310

[b39] Takahashi N. Acid-neutralizing activity during amino acid fermentation by *Porphyromonas gingivalis*, *Prevotella intermedia* and *Fusobacterium nucleatum*. Oral Microbiol. Immunol. 18, 109–113 (2003). 12654101 10.1034/j.1399-302x.2003.00054.x

[b40] Diaz P. I., Zilm P. S. & Rogers A. H. Fusobacterium nucleatum supports the growth of *Porphyromonas gingivalis* in oxygenated and carbon-dioxide-depleted environments. *Microbiology*. 148, 467–472 (2002). 10.1099/00221287-148-2-467 11832510

[b41] Saito A. *et al.* *Fusobacterium nucleatum* enhances invasion of human gingival epithelial and aortic endothelial cells by *Porphyromonas gingivalis*. FEMS Immunol. Med. Microbiol. 54, 349–355 (2008). 19049647 10.1111/j.1574-695X.2008.00481.x

[b42] Dahlen G. & Leonhardt A. A new checkerboard panel for testing bacterial markers in periodontal disease. Oral Microbiol. Immunol. 21, 6–11 (2006). 16390335 10.1111/j.1399-302X.2005.00243.x

[b43] Kumar P. S. *et al.* Changes in periodontal health status are associated with bacterial community shifts as assessed by quantitative 16S cloning and sequencing. J. Clin. Microbiol. 44, 3665–3673 (2006). 17021095 10.1128/JCM.00317-06 PMC1594761

[b44] Aruni A. W., Roy F. & Fletcher H. M. *Filifactor alocis* has virulence attributes that can enhance its persistence under oxidative stress conditions and mediate invasion of epithelial cells by *Porphyromonas gingivalis*. Infect. Immun. 79, 3872–3886 (2011). 21825062 10.1128/IAI.05631-11 PMC3187275

[b45] Moffatt C. E., Whitmore S. E., Griffen A. L., Leys E. J. & Lamont R. J. *Filifactor alocis* interactions with gingival epithelial cells. Mol. Oral Microbiol. 26, 365–373 (2011). 22053964 10.1111/j.2041-1014.2011.00624.x PMC3248241

[b46] Wang Q., Wright C. J., Dingming H., Uriarte S. M. & Lamont R. J. Oral Community interactions of *Filifactor alocis in vitro*. PLoS One 8, e76271 (2013). 24098460 10.1371/journal.pone.0076271 PMC3789735

[b47] Haffajee A. D., Teles R. P. & Socransky S. S. Association of *Eubacterium nodatum* and *Treponema denticola* with human periodontitis lesions. Oral Microbiol. Immunol. 21, 269–282 (2006). 16922925 10.1111/j.1399-302X.2006.00287.x

[b48] Lombardo Bedran T. B., Marcantonio R. A. C., Spin Neto R., Alves Mayer M. P. & Grenier D. Porphyromonas endodontalis in chronic periodontitis: a clinical and microbiological cross-sectional study. J. Oral Microbiol. 4 (2012). 10.3402/jom.v4i0.10123 PMC3253302 22232719

[b49] Inagaki S., Onishi S., Kuramitsu H. K. & Sharma A. *Porphyromonas gingivalis* vesicles enhance attachment, and the Leucine-rich repeat BspA protein is required for invasion of epithelial cells by “*Tannerella forsythia*”. Infect. Immun. 74, 5023–5028 (2006). 16926393 10.1128/IAI.00062-06 PMC1594857

[b50] Yoneda M. *et al.* Mixed infection of *Porphyromonas gingivalis* and *Bacteroides forsythus* in a murine abscess model: involvement of gingipains in a synergistic effect. J. Periodontal Res. 36, 237–243 (2001). 11519697 10.1034/j.1600-0765.2001.036004237.x

[b51] Kuramitsu H. K., He X., Lux R., Anderson M. H. & Shi W. Interspecies interactions within oral microbial communities. Microbiol. Mol. Biol. Rev. 71, 653–670 (2007). 18063722 10.1128/MMBR.00024-07 PMC2168648

[b52] Zhang L., Henson B. S., Camargo P. M. & Wong D. T. The clinical value of salivary biomarkers for periodontal disease. Periodontol. 2000 51, 25–37 (2009). 19878467 10.1111/j.1600-0757.2009.00315.x

[b53] Li M. *et al.* Symbiotic gut microbes modulate human metabolic phenotypes. Proc. Natl. Acad. Sci. USA 105, 2117–2122 (2008). 18252821 10.1073/pnas.0712038105 PMC2538887

[b54] Muyzer G., de Waal E. C. & Uitterlinden A. G. Profiling of complex microbial populations by denaturing gradient gel electrophoresis analysis of polymerase chain reaction-amplified genes coding for 16S rRNA. Appl. Environ. Microbiol. 59, 695–700 (1993). 7683183 10.1128/aem.59.3.695-700.1993 PMC202176

[b55] Wang J. *et al.* Modulation of gut microbiota during probiotics-mediated attenuation of metabolic syndrome in high fat diet-fed mice. ISME J. 10.1038/ismej.2014.99 (2014). PMC4274436 24936764

[b56] DeSantis T. Z. *et al.* NAST: a multiple sequence alignment server for comparative analysis of 16S rRNA genes. Nucleic Acids Res. 34, W394–W399 (2006). 16845035 10.1093/nar/gkl244 PMC1538769

[b57] Li W. & Godzik A. Cd-hit: a fast program for clustering and comparing large sets of protein or nucleotide sequences. Bioinformatics 22, 1658–1659 (2006). 16731699 10.1093/bioinformatics/btl158

[b58] Segata N. *et al.* Metagenomic biomarker discovery and explanation. Genome Biol. 12, R60 (2011). 21702898 10.1186/gb-2011-12-6-r60 PMC3218848

[b59] Schloss P. D. & Handelsman J. Introducing DOTUR, a computer program for defining operational taxonomic units and estimating species richness. Appl. Environ. Microbiol. 71, 1501–1506 (2005). 15746353 10.1128/AEM.71.3.1501-1506.2005 PMC1065144

