# Supplementary material for: A Filifactor alocis-centered co-occurrence group associates with periodontitis across different oral habitats
Source: Sci Rep. 2015 Mar 12;5:9053. doi: 10.1038/srep09053 (PMC4356962; doi:10.1038/srep09053)
Supplement: Supplementary Information [file srep09053-s1.pdf]

A *Filifactor alocis*-centered co-occurrence group associates with periodontitis across different oral habitats

Hui Chen[1], Ying Liu[2], Menghui Zhang[1], Guoyang Wang[3], Zhengnan Qi[2], Laura Bridgewater[4], Liping Zhao[1,3], Zisheng Tang[2*] and Xiaoyan Pang[1*]

Files in this data supplement

Supplementary Table S1

Supplementary Table S2

Supplementary Table S3

Supplementary Table S4

Supplementary Table S5

Supplementary Table S6

Supplementary Table S7

Supplementary Figure S1

Supplementary Figure S2

Supplementary Figure S3

Supplementary Tables and Figures

Supplementary Table S1 Diversity indices of the microbiota in five oral habitat sample groups. Values are shown as mean ± SD. Diversity indices marked with different superscript letters are significantly different from each other (ANOVA, P<0.05).

	H-Sal	P-Sal	H-Sup	P-Sup	P-Sub
Shannon diversity index	3.48±0.41[c]	3.81±0.33[b]	3.85±0.22[b]	4.10±0.20[a]	3.80±0.32[b]
Simpson's diversity index	0.93±0.04[b]	0.95±0.02[a]	0.96±0.01[a]	0.97±0.01[a]	0.93±0.05[b]
Rarefaction	113.37±22.61[d]	139.75±21.90[b,c]	127.70±16.65[c]	154.82±18.53[a]	151.80±13.96[a,b]
Chao1	178.91±33.10[d]	220.18±30.80[b,c]	205.14±31.10[c]	253.83±46.48[a]	231.71±24.22[a,b]

Supplementary Table S2 Oral taxa commonly shared in the microbiota of saliva, supra and subgingival plaque in all individuals.

**Taxa**	**Relative contribution (%)** [1]	**Relative abundance (%) (median, range)**				
		**H-Sal**	**P-Sal**	**H-Sup**	**P-Sup**	**P-Sub**
**Genus level**						
Streptococcus	8.60	17.4, 6.49-31.3	14.4, 3.61-29.7	4.18, 1.88-14.2	3.88, 1.30-8.91	1.33, 0.27-4.16
Leptotrichia	6.87	2.67, 0.83-11.5	2.56, 0.86-8.25	10.4, 1.40-19.6	10.6, 6.66-17.7	4.95, 0.74-16.7
Actinomyces	3.43	3.42, 0.59-9.89	3.39, 1.15-12.1	3.32, 0.69-16.3	2.32, 0.27-13.5	1.76, 0.42-13.9
Prevotella	14.05	10.0, 2.15-34.7	13.8, 3.17-37.8	9.77, 1.11-24.0	16.8, 3.54-36.8	14.2, 3.37-28.3
Porphyromonas	11.40	5.73, 0.94-30.9	9.32, 1.09-21.3	5.57, 0.80-16.4	6.67, 2.01-17.7	26.3, 1.70-53.5
Capnocytophaga	6.97	2.72, 0.37-8.51	1.50, 0.40-7.87	14.8, 7.32-27.6	9.05, 6.47-27.6	3.52, 1.37-15.6
Fusobacterium	4.22	2.57, 0.67-5.88	3.21, 1.05-6.25	3.44, 1.06-7.90	4.79, 1.56-7.95	6.25, 0.73-12.2
TM7_genera_incertae_sedis	1.38	0.71, 0.21-2.66	1.06, 0.22-2.97	1.05, 0.19-4.48	1.73, 0.14-5.73	1.44, 0.24-3.34
Clostridium XlVa	1.05	0.96, 0.41-4.54	1.16, 0.08-6.41	0.65, 0.09-3.59	0.97, 0.28-2.60	0.50, 0.05-2.70
**OTU level**						
OTU0039: *Streptococcus* sp. HOT_734 [HOT_707 HOT_677 HOT_638]	5.49	10.8, 1.43-27.1	8.60, 2.18-24.7	2.89, 0.70-10.7	2.07, 0.37-6.37	0.87, 0.20-2.42
OTU0202: *Leptotrichia buccalis* HOT_563	3.45	1.35, 0.19-5.48	1.29, 0.30-3.83	5.36, 0.70-11.4	5.37, 2.96-10.1	2.85, 0.31-6.81
OTU0288: *Prevotella* sp. HOT_317	2.98	0.45, 0.04-2.24	1.10, 0.16-8.11	3.43, 0.13-7.47	5.22, 1.09-11.0	3.13, 0.74-12.4

[1] Relative contribution of a genus or OTU was calculated as percentage of the total reads belonging to this genus or OTU.

Supplementary Table S3 List of taxa with significant differences between two studied cohorts or different intraoral habitats.

Taxa	Relative abundance (%) (median, range)					P values assessed by Mann-Whitney U test					
	H-Sal	P-Sal	H-Sup	P-Sup	P-Sub	H-Sal vs. P-Sal	H-Sup vs. P-Sup	H-Sal vs. H-Sup	P-Sal vs. P-Sup	P-Sup vs. P-Sub	P-Sal vs. P-Sub
Phylum level											
Bacteroidetes	26.4, 6.45-52.1	31.5, 10.6-57.2	39.2, 15.4-51.4	44.2, 22.1-57.2	52.7, 35.4-64.4	0.110	0.011	5.10E-05	2.35E-05	6.98E-05	5.71E-09
Firmicutes	33.6, 15.0-47.9	33.3, 21.9-52.2	13.7, 6.02-23.5	18.3, 9.64-25.2	16.6, 11.2-23.0	0.433	0.014	2.74E-10	4.29E-11	0.506	3.88E-11
Proteobacteria	19.0, 1.63-49.0	10.7, 0.51-31.9	18.0, 4.02-35.5	10.4, 3.83-20.7	4.15, 1.37-14.7	0.002	1.29E-04	0.408	0.544	8.51E-07	1.44E-06
Fusobacteria	5.42, 2.51-15.4	6.36, 2.59-12.9	14.6, 5.64-23.3	16.5, 10.3-24.7	12.6, 3.13-20.8	0.128	0.329	9.67E-09	1.86E-10	0.007	3.13E-07
Actinobacteria	9.65, 1.63-26.7	7.33, 2.27-23.1	8.87, 2.08-32.4	6.74, 1.16-19.3	3.94, 1.23-17.9	0.460	0.043	0.544	0.268	0.043	1.54E-04
Spirochaetes	0.38, 0-4.80	1.57, 0.12-5.51	0.65, 0-3.49	1.38, 0.15-4.61	4.61, 1.24-10.8	1.21E-05	0.007	0.082	0.636	2.13E-09	2.71E-08
TM7	0.71, 0.21-2.66	1.06, 0.22-2.97	1.05, 0.19-4.48	1.73, 0.14-5.73	1.44, 0.24-3.34	0.183	0.035	0.139	0.012	0.198	0.132
SR1	0.11, 0-1.40	0.18, 0-1.69	0.07, 0-0.75	0.06, 0-0.48	0.04, 0-0.35	0.253	0.810	0.425	0.010	0.103	2.32E-05
Synergistetes	0, 0-0.18	0, 0-0.38	0, 0-0.38	0.02, 0-0.85	0.19, 0-1.18	0.398	0.292	0.519	0.437	1.42E-04	1.77E-05
Tenericutes	0, 0-0.33	0.09, 0-1.26	0, 0-0.14	0, 0-0.09	0.11, 0-0.68	4.40E-04	0.865	0.273	1.41E-07	1.22E-05	0.760
Cyanobacteria/Chloroplast	0, 0-0.13	0, 0-0.53	0, 0-0.02	0, 0-0	0, 0-0	0.727	0.317	0.039	0.005	1.000	0.005
Chloroflexi	0, 0-0	0, 0-0.05	0, 0-0	0, 0-0.07	0, 0-0.15	0.040	0.040	1.000	0.861	0.050	0.027
Genus level											
Prevotella	10.0, 2.15-34.7	13.8, 3.17-37.8	9.77, 1.11-24.0	16.8, 3.54-36.8	14.2, 3.37-28.3	0.344	1.21E-04	0.337	0.183	0.046	0.802
Porphyromonas	5.73, 0.94-30.9	9.32, 1.09-21.3	5.57, 0.80-16.4	6.67, 2.01-17.7	26.3, 1.70-53.5	0.035	0.460	0.848	0.086	5.31E-08	7.32E-07
Streptococcus	17.4, 6.49-31.3	14.4, 3.61-29.7	4.18, 1.88-14.2	3.88, 1.30-8.91	1.33, 0.27-4.16	0.110	0.193	3.64E-10	3.31E-10	5.82E-07	4.29E-11
Neisseria	12.3, 0.76-37.9	6.82, 0.04-22.6	7.52, 0.72-20.2	3.68, 0.11-11.7	1.15, 0-3.78	0.006	0.015	0.001	0.026	1.14E-04	1.11E-07
Capnocytophaga	2.72, 0.37-8.51	1.50, 0.40-7.87	14.8, 7.32-27.6	9.05, 6.47-27.6	3.52, 1.37-15.6	0.055	9.77E-04	3.88E-11	6.37E-11	1.06E-08	5.66E-06
Leptotrichia	2.67, 0.83-11.5	2.56, 0.86-8.25	10.4, 1.40-19.6	10.6, 6.66-17.7	4.95, 0.74-16.7	0.595	0.988	2.10E-08	1.39E-10	6.97E-06	1.21E-04
Fusobacterium	2.57, 0.67-5.88	3.21, 1.05-6.25	3.44, 1.06-7.90	4.79, 1.56-7.95	6.25, 0.73-12.2	0.139	0.024	0.011	0.004	0.007	4.91E-06
Actinomyces	3.42, 0.59-9.89	3.39, 1.15-12.1	3.32, 0.69-16.3	2.32, 0.27-13.5	2.32, 0.27-13.5	0.918	0.079	0.712	0.098	0.193	0.001
Selenomonas	0.42, 0-5.44	0.80, 0.06-6.35	2.37, 0.09-8.05	4.48, 0.69-14.5	3.58, 0.94-10.8	0.056	0.003	5.27E-06	4.49E-08	0.069	5.82E-07
Veillonella	3.86, 0.78-15.0	3.62, 0.63-11.5	1.88, 0-7.22	2.08, 0.14-4.51	0.61, 0.09-2.49	0.425	1.000	7.90E-05	0.001	2.86E-05	1.94E-09

Treponema	**0.32, 0-4.80**	**1.55, 0.12-5.51**	**0.65, 0-3.49**	**1.38, 0.15-4.61**	**4.57, 1.24-10.8**	**1.29E-05**	**0.007**	**0.059**	**0.668**	**2.33E-09**	**2.49E-08**
Rothia	**4.41, 0.75-15.4**	**2.90, 0.55-10.8**	**0.69, 0-9.83**	**0.15, 0-1.87**	**0.07, 0-1.17**	**0.101**	**1.20E-04**	**1.53E-07**	**5.73E-10**	**0.051**	**5.22E-11**
Corynebacterium	**0.20, 0-3.32**	**0.32, 0-1.83**	**3.47, 0.80-15.6**	**1.84, 0.42-7.49**	**0.60, 0.18-3.61**	**0.717**	**0.016**	**1.76E-09**	**5.18E-07**	**0.001**	**0.003**
Granulicatella	**2.66, 0.87-6.73**	**3.09, 0.18-11.9**	**0.31, 0-1.03**	**0.10, 0-1.39**	**0.05, 0-0.48**	**0.121**	**0.018**	**3.83E-11**	**7.67E-11**	**0.015**	**3.24E-11**
TM7_genera_incertae_sedis	**0.71, 0.21-2.66**	**1.06, 0.22-2.97**	**1.05, 0.19-4.48**	**1.73, 0.14-5.73**	**1.44, 0.24-3.34**	**0.183**	**0.035**	**0.139**	**0.012**	**0.198**	**0.132**
Haemophilus	**2.96, 0.38-7.43**	**1.61, 0-6.09**	**0.42, 0-3.99**	**0.11, 0-2.01**	**0.02, 0-0.44**	**0.002**	**0.011**	**4.13E-08**	**1.05E-06**	**0.006**	**1.56E-09**
Clostridium XlVa	**0.96, 0.41-4.54**	**1.16, 0.08-6.41**	**0.65, 0.09-3.59**	**0.97, 0.28-2.60**	**0.50, 0.05-2.70**	**1.000**	**0.030**	**0.016**	**0.745**	**0.012**	**0.011**
Gemella	**1.55, 0.06-6.30**	**1.87, 0.09-5.29**	**0.32, 0-1.38**	**0.28, 0-1.45**	**0.15, 0-0.63**	**0.544**	**0.496**	**7.98E-08**	**1.66E-07**	**0.066**	**3.62E-09**
Tannerella	**0.06, 0-0.87**	**0.41, 0-2.44**	**0.07, 0-1.61**	**0.28, 0-1.78**	**2.57, 0.05-10.1**	**6.94E-04**	**0.003**	**0.915**	**0.399**	**2.79E-09**	**2.10E-08**
Filifactor	**0.07, 0-1.90**	**0.78, 0-2.43**	**0.06, 0-0.56**	**0.20, 0-1.13**	**2.00, 0.10-5.02**	**1.34E-04**	**0.003**	**0.677**	**0.002**	**9.66E-09**	**1.38E-05**
Peptostreptococcus	**0.43, 0-2.33**	**0.93, 0.18-4.38**	**0.08, 0-1.18**	**0.26, 0-1.69**	**0.30, 0-2.07**	**7.10E-04**	**0.004**	**7.55E-06**	**1.47E-05**	**0.243**	**9.50E-05**
Campylobacter	**0.34, 0-2.47**	**0.27, 0.10-0.71**	**0.75, 0.14-3.45**	**0.86, 0.21-2.11**	**0.56, 0.08-1.60**	**0.712**	**0.425**	**1.14E-04**	**2.29E-08**	**0.006**	**5.10E-05**
Aggregatibacter	**0.21, 0-1.28**	**0.38, 0-1.18**	**0.80, 0-3.55**	**0.56, 0-2.88**	**0.10, 0-0.89**	**0.120**	**0.176**	**7.03E-04**	**0.137**	**2.73E-04**	**3.63E-04**
Soonwooa	**0.37, 0-2.18**	**0.22, 0-2.16**	**0.34, 0-6.49**	**0.22, 0-2.25**	**0.05, 0-0.77**	**0.080**	**0.089**	**0.684**	**0.723**	**0.003**	**1.42E-04**
Kingella	**0.11, 0-0.71**	**0.06, 0-1.21**	**0.67, 0.05-1.88**	**0.31, 0.06-3.31**	**0.15, 0-4.25**	**0.447**	**0.002**	**2.25E-08**	**1.11E-04**	**0.003**	**0.237**
Cardiobacterium	**0.06, 0-0.55**	**0.05, 0-0.19**	**0.77, 0.07-3.83**	**0.35, 0-2.08**	**0.20, 0-1.41**	**0.467**	**5.12E-04**	**2.01E-09**	**3.04E-07**	**0.018**	**4.19E-05**
Dialister	**0.09, 0-0.99**	**0.20, 0-1.64**	**0.24, 0-1.09**	**0.27, 0-1.59**	**0.48, 0.05-2.18**	**0.063**	**0.773**	**0.005**	**0.294**	**0.003**	**1.45E-04**
Eubacterium	**0.12, 0-0.93**	**0.54, 0-1.58**	**0.04, 0-0.38**	**0.11, 0-0.52**	**0.61, 0.15-1.26**	**7.99E-05**	**0.098**	**0.023**	**1.10E-06**	**9.30E-09**	**0.249**
Schwartzia	**0, 0-0.29**	**0.05, 0-0.40**	**0.09, 0-0.48**	**0.30, 0-1.33**	**0.38, 0-2.17**	**0.007**	**1.24E-03**	**3.06E-04**	**1.91E-05**	**0.287**	**1.10E-07**
Parvimonas	**0.06, 0-0.46**	**0.25, 0-2.09**	**0.05, 0-0.31**	**0.05, 0-0.47**	**0.11, 0-1.48**	**5.50E-04**	**0.377**	**0.080**	**8.89E-05**	**0.028**	**0.031**
SR1_genera_incertae_sedis	**0.11, 0-1.40**	**0.18, 0-1.69**	**0.07, 0-0.75**	**0.06, 0-0.48**	**0.04, 0-0.35**	**0.253**	**0.810**	**0.425**	**0.010**	**0.103**	**2.32E-05**
Hallella	**0.02, 0-0.31**	**0.09, 0-0.71**	**0.05, 0-0.73**	**0.11, 0-3.13**	**0.15, 0-1.34**	**0.020**	**0.052**	**0.177**	**0.064**	**0.941**	**0.065**
Peptostreptococcaceae_incertae_sedis	**0.05, 0-0.20**	**0.09, 0-0.31**	**0.05, 0-0.91**	**0.11, 0-0.92**	**0.14, 0-0.83**	**0.468**	**0.206**	**0.553**	**0.140**	**0.253**	**0.006**
Olsenella	**0, 0-0.38**	**0.09, 0-0.49**	**0.04, 0-0.42**	**0.06, 0-0.64**	**0.20, 0-1.45**	**0.018**	**0.117**	**0.454**	**0.922**	**0.001**	**0.001**
Solobacterium	**0.12, 0-1.13**	**0.19, 0.04-1.66**	**0, 0-0.22**	**0, 0-0.25**	**0, 0-0.21**	**0.139**	**0.652**	**7.20E-05**	**4.88E-08**	**0.987**	**8.77E-08**
Limnohabitans	**0, 0-0.11**	**0, 0-0.41**	**0.05, 0-0.96**	**0.05, 0-2.53**	**0, 0-1.15**	**0.041**	**0.579**	**6.71E-04**	**0.037**	**0.083**	**0.817**
Eikenella	**0, 0-0.35**	**0, 0-0.42**	**0.14, 0-1.51**	**0.11, 0-0.60**	**0.06, 0-0.34**	**0.603**	**0.198**	**1.18E-06**	**1.14E-05**	**0.126**	**5.87E-04**
Atopobium	**0.07, 0-1.02**	**0.11, 0-1.40**	**0, 0-0.07**	**0, 0-0.21**	**0, 0-0.15**	**0.284**	**0.370**	**4.99E-04**	**7.13E-05**	**0.664**	**1.23E-05**
Peptococcus	**0, 0-0.27**	**0.12, 0-0.46**	**0, 0-0.13**	**0.02, 0-0.34**	**0.11, 0-0.48**	**3.84E-04**	**0.285**	**0.567**	**0.003**	**0.010**	**0.738**

Megasphaera	**0.05, 0-1.67**	**0.05, 0-1.72**	**0, 0-0.10**	**0, 0-0.35**	**0, 0-0.41**	**0.811**	**0.004**	**2.55E-05**	**0.072**	**0.117**	**0.002**
Mycoplasma	**0, 0-0.26**	**0.09, 0-1.26**	**0, 0-0.14**	**0, 0-0.09**	**0.11, 0-0.68**	**2.04E-04**	**0.637**	**0.306**	**7.96E-08**	**6.26E-06**	**0.715**
Phocaeicola	**0, 0-0.27**	**0, 0-0.12**	**0, 0-0.21**	**0.05, 0-1.38**	**0.13, 0-1.52**	**0.243**	**0.011**	**0.460**	**0.020**	**0.129**	**5.18E-04**
Bacteroides	**0, 0-0.37**	**0, 0-0.65**	**0, 0-0.11**	**0, 0-0.25**	**0.06, 0-0.55**	**0.142**	**0.012**	**0.661**	**0.796**	**0.007**	**0.007**
Anaeroglobus	**0, 0-0.60**	**0, 0-0.35**	**0, 0-0.36**	**0.04, 0-0.37**	**0.10, 0-0.44**	**0.085**	**0.002**	**0.864**	**0.167**	**0.134**	**0.016**
Desulfobulbus	**0, 0-0.25**	**0, 0-0.20**	**0, 0-0.07**	**0, 0-0.16**	**0.10, 0-0.63**	**0.047**	**0.064**	**0.596**	**0.475**	**0.003**	**0.013**
Johnsonella	**0, 0-0.13**	**0, 0-0.12**	**0, 0-0.75**	**0, 0-0.30**	**0.02, 0-1.46**	**0.460**	**1.000**	**0.008**	**0.059**	**0.277**	**0.003**
Shuttleworthia	**0, 0-0.32**	**0.07, 0-0.52**	**0, 0-0.09**	**0, 0-0.42**	**0, 0-0.34**	**1.11E-04**	**0.324**	**0.283**	**1.11E-04**	**0.886**	**2.07E-04**
Dysgonomonas	**0, 0-0.07**	**0, 0-0.10**	**0, 0-0.08**	**0, 0-1.45**	**0, 0-0.69**	**0.622**	**0.010**	**0.379**	**0.006**	**0.648**	**0.013**
Lachnospiracea_incertae_sedis	**0, 0-0.19**	**0, 0-0.27**	**0, 0-0**	**0, 0-0.23**	**0.05, 0-0.59**	**0.009**	**6.35E-04**	**0.021**	**0.210**	**0.035**	**0.351**
Butyrivibrio	**0, 0-0.61**	**0, 0-0.50**	**0, 0-0**	**0, 0-0.27**	**0, 0-0.36**	**0.797**	**0.154**	**1.39E-04**	**0.011**	**1.000**	**0.010**
Moryella	**0, 0-0.20**	**0, 0-0.20**	**0, 0-0.13**	**0, 0-0.11**	**0.05, 0-0.18**	**0.007**	**0.185**	**0.175**	**0.004**	**6.21E-05**	**0.278**
Mogibacterium	**0, 0-0.31**	**0.05, 0-0.28**	**0, 0-0**	**0, 0-0.10**	**0, 0-0.12**	**7.82E-04**	**0.040**	**0.001**	**4.20E-06**	**0.157**	**2.12E-04**
Streptobacillus	**0, 0-0.05**	**0, 0-0.52**	**0, 0-0.07**	**0, 0-0.06**	**0, 0-0**	**0.006**	**0.185**	**0.330**	**0.010**	**0.040**	**1.39E-04**

Enriched in periodontitis patients

Enriched in healthy populations

Enriched in saliva

Enriched in supragingival plaque

Enriched in subgingival plaque

Supplementary Table S4 Phylogeny of the 110 key OTUs identified by two-step RDA, which showed significant differences between periodontitis patients and healthy controls.

**OTU name**	**Domain**	**Phylum**	**Class**	**Order**	**Family**	**Genus**	**Nearest neighbors (based HOMD)**
OTU0089	Bacteria	*Actinobacteria*	*Actinobacteria*	*Actinomycetales*	*Actinomycetaceae*	*Actinomyces*	*Actinomyces cardiffensis*; HOT_850; GU470888 (99.4%)
OTU0054	Bacteria	*Bacteroidetes*	*Bacteroidia*	*Bacteroidales*	*Porphyromonadaceae*	*Porphyromonas*	*Porphyromonas gingivalis*; HOT_619; X73964 (100%)
OTU0096	Bacteria	*Bacteroidetes*	*Bacteroidia*	*Bacteroidales*	*Porphyromonadaceae*	*Porphyromonas*	*Porphyromonas endodontalis*; HOT_273; L16491 (100%)
OTU0204	Bacteria	*Bacteroidetes*	*Bacteroidia*	*Bacteroidales*	*Porphyromonadaceae*	*Tannerella*	*Tannerella forsythia*; HOT_613; L16495 (100%)
OTU0200	Bacteria	*Bacteroidetes*	*Bacteroidia*	*Bacteroidales*	*Prevotellaceae*	*Prevotella*	*Prevotella* sp.; HOT_304; AY005065 (100%)
OTU0290	Bacteria	*Bacteroidetes*	*Bacteroidia*	*Bacteroidales*	*Prevotellaceae*	*Prevotella*	*Prevotella* sp.; HOT_526; DQ003633 (100%)
OTU0211	Bacteria	*Bacteroidetes*	*Bacteroidia*	*Bacteroidales*	*Prevotellaceae*	*Prevotella*	*Prevotella* sp.; HOT_443; AY331415 (99.4%)
OTU0456	Bacteria	*Bacteroidetes*	*Bacteroidia*	*Bacteroidales*	*Prevotellaceae*	*Prevotella*	*Prevotella loescheii*; HOT_658; L16481 (96.2%)
OTU0304	Bacteria	*Bacteroidetes*					*Bacteroidetes* [G-3] sp.; HOT_280; AY005071 (100%)
OTU0177	Bacteria	*Firmicutes*	*Clostridia*	*Clostridiales*	*Eubacteriaceae*	*Eubacterium*	*Eubacterium* [XI][G-6] *nodatum*; HOT_694; Z36274 (100%)
OTU0198	Bacteria	*Firmicutes*	*Clostridia*	*Clostridiales*	*Lachnospiraceae*	*Lachnospiracea_incertae_sedis*	*Moryella* sp.; HOT_419; AY278618 (93.4%)
OTU0308	Bacteria	*Firmicutes*	*Clostridia*	*Clostridiales*	*Lachnospiraceae*		*Lachnospiraceae* [G-8] sp.; HOT_500; AY349389 (99.3%)
OTU0263	Bacteria	*Firmicutes*	*Clostridia*	*Clostridiales*	*Peptostreptococcaceae*	*Filifactor*	*Filifactor alocis*; HOT_539; AJ006962 (100%)
OTU0115	Bacteria	*Firmicutes*	*Clostridia*	*Clostridiales*	*Peptostreptococcaceae*	*Peptostreptococcus*	*Peptostreptococcus stomatis*; HOT_112; GQ422715 (100%)
OTU0287	Bacteria	*Firmicutes*	*Clostridia*	*Clostridiales*			*Johnsonella* sp.; HOT_166; AF287774 (99.3%)
OTU0413	Bacteria	*Firmicutes*	*Clostridia*	*Clostridiales*			*Peptostreptococcaceae* [XI][G-4] sp.; HOT_369; AF481212 (100%)
OTU0312	Bacteria	*Firmicutes*	*Clostridia*	*Clostridiales*			*Peptostreptococcaceae* [XI][G-3] sp.; HOT_382; HE681251 (100%)
OTU0463	Bacteria	*Firmicutes*	*Clostridia*	*Clostridiales*			*Peptostreptococcaceae* [XI][G-3] sp.; HOT_382; HE681251 (89.3%)
OTU0309	Bacteria	*Firmicutes*					*Mollicutes* [G-2] sp.; HOT_906; KC203059 (100%)
OTU0146	Bacteria	*Fusobacteria*	*Fusobacteria*	*Fusobacteriales*	*Leptotrichiaceae*		*Leptotrichiaceae* [G-1] sp.; HOT_220; AF385542 (100%)
OTU0223	Bacteria						*Fretibacterium* sp.; HOT_452; AY349370 (100%)<br>*Fretibacterium sp.*; HOT_359; AY005447 (100%)
OTU0126	Bacteria	*Actinobacteria*	*Actinobacteria*	*Coriobacteriales*	*Coriobacteriaceae*	*Olsenella*	*Olsenella uli*; HOT_038; AF292373 (100%)
OTU0655	Bacteria	*Bacteroidetes*	*Bacteroidia*	*Bacteroidales*	*Porphyromonadaceae*	*Porphyromonas*	*Porphyromonas* sp.; HOT_278; AF385560 (98.1%)<br>*Porphyromonas* sp.; HOT_277; AF385555 (98.1%)
OTU0294	Bacteria	*Bacteroidetes*	*Bacteroidia*	*Bacteroidales*	*Prevotellaceae*	*Prevotella*	*Prevotella* sp.; HOT_526; DQ003633 (99.4%)
OTU0288	Bacteria	*Bacteroidetes*	*Bacteroidia*	*Bacteroidales*	*Prevotellaceae*	*Prevotella*	*Prevotella shahii*; HOT_795; AB108825 (98.7%)

OTU0196	Bacteria	*Bacteroidetes*	*Bacteroidia*	*Bacteroidales*	*Prevotellaceae*	*Prevotella*	*Prevotella nigrescens*; HOT_693; X73963 (98.7%)
OTU0209	Bacteria	*Bacteroidetes*	*Bacteroidia*	*Bacteroidales*	*Prevotellaceae*	*Prevotella*	*Alloprevotella tannerae*; HOT_466; AJ005634 (98.7%)
OTU0462	Bacteria	*Bacteroidetes*	*Bacteroidia*	*Bacteroidales*	*Prevotellaceae*	*Prevotella*	*Prevotella loescheii*; HOT_658; L16481 (94.9%)
OTU0338	Bacteria	*Bacteroidetes*					*Bacteroidetes* [G-3] sp.; HOT_503; DQ003613 (100%)
OTU0113	Bacteria	*Firmicutes*	*Bacilli*	*Lactobacillales*	*Streptococcaceae*	*Streptococcus*	*Streptococcus constellatus*; HOT_576; AF104676 (100%)
OTU0085	Bacteria	*Firmicutes*	*Clostridia*	*Clostridiales*	*Clostridiales_Incertae Sedis XI*	*Parvimonas*	*Parvimonas micra*; HOT_111; AY323523 (100%)
OTU0222	Bacteria	*Firmicutes*	*Clostridia*	*Clostridiales*	*Clostridiales_Incertae Sedis XI*	*Sporanaerobacter*	*Peptostreptococcaceae* [XIII][G-2] sp.; HOT_790; AY134899 (100%)
OTU0217	Bacteria	*Firmicutes*	*Clostridia*	*Clostridiales*	*Clostridiales_Incertae Sedis XI*		*Peptostreptococcaceae* [XIII][G-1] sp.; HOT_113; AF287766 (100%)
OTU0103	Bacteria	*Firmicutes*	*Clostridia*	*Clostridiales*	*Clostridiales_Incertae Sedis XIII*	*Mogibacterium*	*Mogibacterium neglectum*; HOT_691; AB037875 (100%)<br>*Mogibacterium diversum*; HOT_593; AB037874 (100%)<br>*Mogibacterium vescum*; HOT_008; AB021702 (100%)
OTU0099	Bacteria	*Firmicutes*	*Clostridia*	*Clostridiales*	*Eubacteriaceae*	*Eubacterium*	*Eubacterium* [XI][G-3] *brachy*; HOT_557; U13038 (100%)
OTU0273	Bacteria	*Firmicutes*	*Clostridia*	*Clostridiales*	*Eubacteriaceae*	*Eubacterium*	*Eubacterium* [XI][G-5] *saphenum*; HOT_759; U65987 (99.3%)
OTU0056	Bacteria	*Firmicutes*	*Clostridia*	*Clostridiales*	*Incertae Sedis XI*	*Murdochiella*	*Parvimonas micra*; HOT_111; AY323523 (89.9%)
OTU0272	Bacteria	*Firmicutes*	*Clostridia*	*Clostridiales*	*Lachnospiraceae*	*Catonella*	*Catonella morbi*; HOT_165; DQ003629 (97.1%)
OTU0134	Bacteria	*Firmicutes*	*Clostridia*	*Clostridiales*	*Lachnospiraceae*	*Shuttleworthia*	*Shuttleworthia satelles*; HOT_095; AF399956 (100%)
OTU0356	Bacteria	*Firmicutes*	*Clostridia*	*Clostridiales*	*Peptococcaceae 1*	*Peptococcus*	*Peptococcus* sp.; HOT_168; AF287780 (98.1%)
OTU0353	Bacteria	*Firmicutes*	*Erysipelotrichia*	*Erysipelotrichales*	*Erysipelotrichaceae*	*Solobacterium*	*Solobacterium moorei*; HOT_678; AY044915 (97.5%)
OTU0255	Bacteria	*Firmicutes*	*Negativicutes*	*Selenomonadales*	*Veillonellaceae*	*Schwartzia*	*Veillonellaceae* [G-1] sp.; HOT_483; AY349410 (100%)
OTU0432	Bacteria	*Fusobacteria*	*Fusobacteria*	*Fusobacteriales*	*Leptotrichiaceae*	*Leptotrichia*	*Leptotrichia goodfellowii*; HOT_845; AY029807 (100%)
OTU0630	Bacteria	*Spirochaetes*	*Spirochaetes*	*Spirochaetales*	*Spirochaetaceae*	*Treponema*	*Treponema socranskii ss socranskii*; HOT_769; AF033306 (89.5%)
OTU0354	Bacteria	*Spirochaetes*	*Spirochaetes*	*Spirochaetales*	*Spirochaetaceae*	*Treponema*	*Treponema* sp.; HOT_270; GQ422733 (100%)
OTU0259	Bacteria	*Spirochaetes*	*Spirochaetes*	*Spirochaetales*	*Spirochaetaceae*	*Treponema*	*Treponema* sp.; HOT_517; DQ003624 (100%)<br>*Treponema* sp.; HOT_256; AF182835 (100%)<br>*Treponema* sp.; HOT_253; AF182838 (100%)
OTU0265	Bacteria	*Spirochaetes*	*Spirochaetes*	*Spirochaetales*	*Spirochaetaceae*	*Treponema*	*Treponema* sp.; HOT_237; AF056337 (100%)
OTU0274	Bacteria	*Spirochaetes*	*Spirochaetes*	*Spirochaetales*	*Spirochaetaceae*	*Treponema*	*Treponema denticola*; HOT_584; M71236 (100%)
OTU0276	Bacteria	*Spirochaetes*	*Spirochaetes*	*Spirochaetales*	*Spirochaetaceae*	*Treponema*	*Treponema* sp.; HOT_237; AY341822 (99.4%)<br>*Treponema* sp.; HOT_234; AF023056 (99.4%)
OTU0298	Bacteria	*Spirochaetes*	*Spirochaetes*	*Spirochaetales*	*Spirochaetaceae*	*Treponema*	*Treponema socranskii ss socranskii*; HOT_769; AF033306 (89.4%)
OTU0307	Bacteria	*Spirochaetes*	*Spirochaetes*	*Spirochaetales*	*Spirochaetaceae*	*Treponema*	*Treponema denticola*; HOT_584; AF023030 (99.4%)
OTU0366	Bacteria	*Spirochaetes*	*Spirochaetes*	*Spirochaetales*	*Spirochaetaceae*	*Treponema*	*Treponema* sp.; HOT_234; AF023056 (100%)

OTU0475	Bacteria	*Spirochaetes*	*Spirochaetes*	*Spirochaetales*	*Spirochaetaceae*	*Treponema*	*Treponema* sp.; HOT_237; AY341822 (98.8%)<br>*Treponema* sp.; HOT_234; AF023056 (98.8%)
OTU0489	Bacteria	*Spirochaetes*	*Spirochaetes*	*Spirochaetales*	*Spirochaetaceae*	*Treponema*	*Treponema* sp.; HOT_254; AF182836 (96.9%)
OTU0160	Bacteria	*Tenericutes*	*Mollicutes*	*Mycoplasmatales*	*Mycoplasmataceae*	*Mycoplasma*	*Mycoplasma faucium*; HOT_606; AF125590 (100%)
OTU0236	Bacteria						*Fretibacterium* sp.; HOT_452; AY349370 (99.3%)<br>*Fretibacterium* sp.; HOT_359; AY005447 (99.3%)
OTU0174	Bacteria	*Bacteroidetes*	*Bacteroidia*	*Bacteroidales*	*Prevotellaceae*	*Prevotella*	*Prevotella* sp.; HOT_299; AY005064 (99.4%)
OTU0048	Bacteria	*Firmicutes*	*Clostridia*	*Clostridiales*	*Lachnospiraceae*	*Clostridium XlVa*	*Oribacterium* sp.; HOT_108; AF287770 (100%)
OTU0186	Bacteria	*Proteobacteria*	*Betaproteobacteria*	*Neisseriales*	*Neisseriaceae*	*Neisseria*	*Neisseria pharyngis*; HOT_729; AJ239281 (99.4%)
OTU0392	Bacteria	*Proteobacteria*	*Betaproteobacteria*	*Neisseriales*	*Neisseriaceae*	*Neisseria*	*Neisseria subflava*; HOT_476; AJ239291 (98.8%)
OTU0153	Bacteria	*Proteobacteria*	*Epsilonproteobacteria*	*Campylobacterales*	*Campylobacteraceae*	*Campylobacter*	*Campylobacter concisus*; HOT_575; L06977 (99.3%)
OTU0285	Bacteria	*Actinobacteria*	*Actinobacteria*	*Actinomycetales*	*Actinomycetaceae*	*Actinomyces*	*Actinomyces israelii*; HOT_645; X82450 (98.1%)
OTU0305	Bacteria	*Bacteroidetes*	*Bacteroidia*	*Bacteroidales*	*Bacteroidaceae*	*Bacteroides*	*Bacteroides heparinolyticus*; HOT_630; GQ422742 (100%)
OTU0581	Bacteria	*Bacteroidetes*	*Bacteroidia*	*Bacteroidales*	*Porphyromonadaceae*	*Dysgonomonas*	*Bacteroidales* [G-2] sp.; HOT_274; AY005072 (98.7%)
OTU0415	Bacteria	*Bacteroidetes*	*Bacteroidia*	*Bacteroidales*	*Porphyromonadaceae*		*Tannerella* sp.; HOT_916; KC203065 (99.4%)
OTU0171	Bacteria	*Bacteroidetes*	*Bacteroidia*	*Bacteroidales*	*Porphyromonadaceae*		*Tannerella* sp.; HOT_916; KC203065 (97.5%)
OTU0249	Bacteria	*Bacteroidetes*	*Bacteroidia*	*Bacteroidales*	*Prevotellaceae*	*Prevotella*	*Prevotella shahii*; HOT_795; AB108825 (96.2%)
OTU0556	Bacteria	*Bacteroidetes*	*Bacteroidia*	*Bacteroidales*	*Prevotellaceae*	*Prevotella*	*Prevotella intermedia*; HOT_643; L16468 (94.9%)
OTU0173	Bacteria	*Bacteroidetes*	*Bacteroidia*	*Bacteroidales*	*Prevotellaceae*	*Prevotella*	*Prevotella intermedia*; HOT_643; L16468 (98.1%)
OTU0334	Bacteria	*Bacteroidetes*	*Bacteroidia*	*Bacteroidales*	*Prevotellaceae*	*Prevotella*	*Prevotella* sp.; HOT_301; AF385558 (100%)
OTU0615	Bacteria	*Bacteroidetes*	*Bacteroidia*	*Bacteroidales*	*Prevotellaceae*	*Prevotella*	*Prevotella shahii*; HOT_795; AB108825 (97.5%)
OTU0108	Bacteria	*Bacteroidetes*	*Bacteroidia*	*Bacteroidales*	*Prevotellaceae*	*Prevotella*	*Prevotella melaninogenica*; HOT_469; AY323525 (100%)
OTU0179	Bacteria	*Bacteroidetes*	*Bacteroidia*	*Bacteroidales*	*Prevotellaceae*	*Prevotella*	*Prevotella veroralis*; HOT_572; L16473 (99.4%)<br>*Prevotella histicola*; HOT_298; AF385551 (99.4%)
OTU0193	Bacteria	*Bacteroidetes*	*Bacteroidia*	*Bacteroidales*	*Prevotellaceae*	*Prevotella*	*Prevotella histicola*; HOT_298; AF385551 (97.5%)
OTU0228	Bacteria	*Bacteroidetes*	*Bacteroidia*	*Bacteroidales*	*Prevotellaceae*	*Prevotella*	*Prevotella* sp.; HOT_443; AY331415 (100%)
OTU0358	Bacteria	*Bacteroidetes*	*Bacteroidia*	*Bacteroidales*	*Prevotellaceae*	*Prevotella*	*Prevotella micans*; HOT_378; AF481228 (98.7%)
OTU0341	Bacteria	*Bacteroidetes*	*Bacteroidia*	*Bacteroidales*	*Prevotellaceae*		*Prevotella* sp.; HOT_396; AY207050 (96.2%)
OTU0510	Bacteria	*Bacteroidetes*	*Bacteroidia*	*Bacteroidales*			*Tannerella* sp.; HOT_286; AY008308 (96.8%)
OTU0492	Bacteria	*Chloroflexi*	*Anaerolineae*	*Anaerolineales*	*Anaerolineaceae*		*Chloroflexi* [G-1] sp.; HOT_439; AY331414 (100%)
OTU0032	Bacteria	*Firmicutes*	*Bacilli*	*Lactobacillales*	*Streptococcaceae*	*Streptococcus*	*Streptococcus parasanguinis II*; HOT_411; EF399017 (100%)
OTU0088	Bacteria	*Firmicutes*	*Clostridia*	*Clostridiales*	*Lachnospiraceae*	*Clostridium XlVa*	*Lachnoanaerobaculum* sp.; HOT_083; AF385562 (100%)

OTU0350	Bacteria	*Firmicutes*	*Clostridia*	*Clostridiales*	*Lachnospiraceae*		*Lachnospiraceae* [G-5] sp.; HOT_080; AF287772 (100%)
OTU0264	Bacteria	*Firmicutes*	*Clostridia*	*Clostridiales*			*Peptostreptococcaceae* [XI][G-5] sp.; HOT_493; AY349373 (96.4%)
OTU0074	Bacteria	*Firmicutes*	*Negativicutes*	*Selenomonadales*	*Veillonellaceae*	*Centipeda*	*Selenomonas* sp.; HOT_149; AY349403 (100%)
OTU0072	Bacteria	*Firmicutes*	*Negativicutes*	*Selenomonadales*	*Veillonellaceae*	*Selenomonas*	*Selenomonas infelix*; HOT_639; AF287802 (100%)
OTU0437	Bacteria	*Firmicutes*	*Negativicutes*	*Selenomonadales*	*Veillonellaceae*		*Selenomonas sputigena*; HOT_151; GQ422722 (96.3%)
OTU0402	Bacteria	*Firmicutes*					*Treponema denticola*; HOT_584; AF023030 (83.4%)
OTU0138	Bacteria	*Fusobacteria*	*Fusobacteria*	*Fusobacteriales*	*Fusobacteriaceae*	*Fusobacterium*	*Fusobacterium periodonticum*; HOT_201; X55405 (99.3%)
OTU0539	Bacteria	*Fusobacteria*	*Fusobacteria*	*Fusobacteriales*	*Fusobacteriaceae*	*Fusobacterium*	*Fusobacterium naviforme*; HOT_689; AJ006965 (94.3%)<br>*Fusobacterium nucleatum ss. animalis*; HOT_420; X55404 (94.3%)
OTU0503	Bacteria	*Fusobacteria*	*Fusobacteria*	*Fusobacteriales*	*Leptotrichiaceae*	*Leptotrichia*	*Leptotrichia* sp.; HOT_221; AF432138 (96.5%)
OTU0197	Bacteria	*Fusobacteria*	*Fusobacteria*	*Fusobacteriales*	*Leptotrichiaceae*	*Leptotrichia*	*Leptotrichia* sp.; HOT_498; AY349387 (100%)
OTU0270	Bacteria	*Fusobacteria*	*Fusobacteria*	*Fusobacteriales*	*Leptotrichiaceae*	*Leptotrichia*	*Leptotrichia buccalis*; HOT_563; L37788 (99.3%)
OTU1609	Bacteria	*Proteobacteria*	*Betaproteobacteria*	*Neisseriales*	*Neisseriaceae*	*Kingella*	*Neisseria elongata*; HOT_598; L06171 (98.8%)
OTU0424	Bacteria	*Proteobacteria*	*Deltaproteobacteria*	*Desulfovibrionales*			*Desulfovibrio* sp.; HOT_040; AY005035 (94.4%)
OTU0183	Bacteria	*Spirochaetes*	*Spirochaetes*	*Spirochaetales*	*Spirochaetaceae*	*Treponema*	*Treponema medium*; HOT_667; AF023051 (100%)<br>*Treponema vincentii*; HOT_029; AF033309 (100%)
OTU0313	Bacteria	*Spirochaetes*	*Spirochaetes*	*Spirochaetales*	*Spirochaetaceae*	*Treponema*	*Treponema socranskii ss 04*; HOT_769; AF033308 (100%)
OTU0364	Bacteria	*Spirochaetes*	*Spirochaetes*	*Spirochaetales*	*Spirochaetaceae*	*Treponema*	*Treponema socranskii ss paredis*; HOT_769; AF033307 (100%)
OTU0423	Bacteria	*Spirochaetes*	*Spirochaetes*	*Spirochaetales*	*Spirochaetaceae*	*Treponema*	*Treponema maltophilum*; HOT_664; AF023039 (100%)
OTU0058	Bacteria	*TM7*	*TM7_genera_incertae_sedis*	*TM7_genera_incertae_sedis*	*TM7_genera_incertae_sedis*	*TM7_genera_incertae_sedis*	*TM7* [G-5] sp.; HOT_437; AY331416 (100%)
OTU0151	Bacteria	*TM7*	*TM7_genera_incertae_sedis*	*TM7_genera_incertae_sedis*	*TM7_genera_incertae_sedis*	*TM7_genera_incertae_sedis*	*TM7* [G-1] sp.; HOT_347; AY005446 (99.3%)
OTU0226	Bacteria						*Fretibacterium fastidiosum*; HOT_363; AF125202 (100%)
OTU0210	Bacteria	*Actinobacteria*	*Actinobacteria*	*Actinomycetales*	*Corynebacteriaceae*	*Corynebacterium*	*Corynebacterium durum*; HOT_595; Z97069 (100%)
OTU0107	Bacteria	*Actinobacteria*	*Actinobacteria*	*Actinomycetales*	*Micrococcaceae*	*Rothia*	*Rothia dentocariosa*; HOT_587; M59055 (100%)<br>*Rothia aeria*; HOT_188; Y13025 (100%)
OTU0162	Bacteria	*Bacteroidetes*	*Bacteroidia*	*Bacteroidales*	*Porphyromonadaceae*	*Porphyromonas*	*Porphyromonas* sp.; HOT_279; AY008310 (98.7%)
OTU0201	Bacteria	*Bacteroidetes*	*Flavobacteria*	*Flavobacteriales*	*Flavobacteriaceae*	*Capnocytophaga*	*Capnocytophaga granulosa*; HOT_325; U41347 (100%)
OTU0181	Bacteria	*Proteobacteria*	*Betaproteobacteria*	*Burkholderiales*			*Lautropia mirabilis*; HOT_022; X73223 (100%)
OTU0242	Bacteria	*Proteobacteria*	*Betaproteobacteria*	*Burkholderiales*			*Lautropia mirabilis*; HOT_022; AY005030 (100%)
OTU0992	Bacteria	*Proteobacteria*	*Betaproteobacteria*	*Burkholderiales*			*Lautropia mirabilis*; HOT_022; X73223 (95.7%)
OTU1199	Bacteria	*Proteobacteria*	*Betaproteobacteria*	*Burkholderiales*			*Lautropia mirabilis*; HOT_022; X73223 (94.4%)
OTU0199	Bacteria	*Proteobacteria*	*Betaproteobacteria*	*Neisseriales*	*Neisseriaceae*	*Neisseria*	*Neisseria elongata*; HOT_598; L06171 (100%)

Supplementary Table S5 Univariate comparisons of the 110 key OTUs identified by RDA.

OTU name	Relative abundance (%) (median, range)				P values assessed by Mann-Whitney U test	
	H-Sal	P-Sal	H-Sup	P-Sup	H-Sal vs. P-Sal	H-Sup vs. P-Sup
OTU0089	0, 0-0.13	0.05, 0-1.82	0, 0-0.06	0, 0-0.28	7.01E-04	0.001
OTU0054	0.08, 0-7.08	1.65, 0-8.75	0.24, 0-10.7	2.60, 0-12.0	3.11E-04	7.43E-04
OTU0096	0.42, 0-2.91	1.87, 0-11.0	0.20, 0-3.51	0.74, 0-3.63	1.63E-04	0.013
OTU0204	0.06, 0-0.87	0.41, 0-2.40	0.07, 0-1.55	0.25, 0-1.67	8.91E-04	0.018
OTU0200	0, 0-1.96	0.16, 0-0.92	0, 0-0.38	0.07, 0-0.50	0.050	0.005
OTU0290	0, 0-0.17	0, 0-0.53	0, 0-0.38	0.06, 0-1.03	0.002	0.006
OTU0211	0, 0-0.19	0.09, 0-0.96	0.07, 0-0.63	0.26, 0-1.26	2.45E-04	8.50E-05
OTU0456	0, 0-0.05	0, 0-0.16	0, 0-0.42	0.09, 0-0.98	0.005	0.015
OTU0304	0, 0-0.46	0.07, 0-0.63	0, 0-0.19	0.07, 0-0.36	0.001	0.002
OTU0177	0, 0-0.40	0.07, 0-0.57	0, 0-0.10	0, 0-0.26	6.87E-05	0.031
OTU0198	0, 0-0.19	0, 0-0.32	0, 0-0	0, 0-0.29	0.004	3.01E-04
OTU0308	0, 0-0.32	0.12, 0-0.76	0, 0-0.21	0.10, 0-0.56	1.32E-04	0.002
OTU0263	0.07, 0-1.84	0.78, 0-2.43	0.06, 0-0.56	0.17, 0-1.08	1.34E-04	0.006
OTU0115	0.43, 0-2.33	0.93, 0.18-4.37	0.08, 0-1.09	0.21, 0-1.69	7.49E-04	0.005
OTU0287	0, 0-0.14	0.04, 0-1.29	0, 0-0.14	0.05, 0-0.37	1.27E-04	0.008
OTU0413	0, 0-0.50	0.08, 0-0.48	0, 0-0.19	0.04, 0-0.34	2.67E-04	0.026
OTU0312	0, 0-0	0, 0-0.16	0, 0-0	0, 0-0.14	0.003	0.021
OTU0463	0, 0-0	0, 0-0.13	0, 0-0	0, 0-0.05	0.005	0.040
OTU0309	0, 0-0	0, 0-0.53	0, 0-0	0, 0-0.06	0.003	0.011
OTU0146	0, 0-0.12	0, 0-3.47	0, 0-0.05	0, 0-0.59	4.52E-04	0.024
OTU0223	0, 0-0.35	0.05, 0-0.38	0, 0-1.02	0.10, 0-0.39	0.011	0.022
OTU0126	0, 0-0.07	0, 0-0.27	0, 0-0.06	0, 0-0.05	0.005	0.688
OTU0655	0, 0-0	0, 0-0.09	0, 0-0.09	0, 0-0.10	0.021	0.727
OTU0294	0, 0-0	0, 0-0.10	0, 0-0.14	0, 0-0.75	0.005	0.078
OTU0288	0.45, 0.04-2.24	1.10, 0.16-8.11	3.43, 0.13-7.47	5.22, 1.09-11.0	0.002	0.009
OTU0196	0.04, 0-1.46	0.10, 0-1.06	0.37, 0-5.18	0.60, 0-2.30	0.002	0.544
OTU0209	0, 0-0.05	0, 0-0.10	0, 0-0.20	0, 0-0.07	0.004	0.634
OTU0462	0, 0-0.05	0, 0-0.16	0, 0-0.06	0, 0-0.09	0.006	0.943
OTU0338	0, 0-0.09	0, 0-0.30	0, 0-0	0, 0-0.11	0.024	0.154
OTU0113	0, 0-0.46	0.12, 0-0.45	0, 0-0.40	0.05, 0-0.35	9.13E-04	0.140
OTU0085	0.06, 0-0.46	0.25, 0-1.95	0.02, 0-0.31	0.05, 0-0.43	4.58E-04	0.300
OTU0222	0, 0-0.05	0, 0-0.19	0, 0-0.06	0, 0-0.17	0.012	0.213
OTU0217	0, 0-0.11	0, 0-0.53	0, 0-0.21	0, 0-0.11	7.68E-04	0.460
OTU0103	0, 0-0.31	0.05, 0-0.28	0, 0-0	0, 0-0.10	0.001	0.078
OTU0099	0, 0-0.16	0.04, 0-0.33	0, 0-0.28	0, 0-0.16	0.002	0.198
OTU0273	0, 0-0.49	0.09, 0-0.86	0, 0-0.14	0, 0-0.20	0.001	0.409
OTU0056	0, 0-0	0, 0-0.23	0, 0-0	0, 0-0.11	0.005	0.317
OTU0272	0, 0-0.11	0, 0-0.13	0, 0-0	0, 0-0	0.029	1.000
OTU0134	0, 0-0.32	0.07, 0-0.52	0, 0-0.09	0, 0-0.42	1.11E-04	0.324
OTU0356	0, 0-0.12	0.05, 0-0.22	0, 0-0.13	0, 0-0.25	0.014	0.101
OTU0353	0, 0-0.35	0.07, 0-0.43	0, 0-0.09	0, 0-0.06	0.009	0.241

OTU0255	0, 0-0.27	0.07, 0-0.66	0.08, 0-0.39	0.25, 0-1.33	6.99E-04	0.007
OTU0432	0, 0-0.07	0, 0-0.95	0, 0-0.26	0, 0-0.10	0.004	0.966
OTU0630	0, 0-0.09	0, 0-0.09	0, 0-0	0, 0-0	0.029	1.000
OTU0354	0, 0-0.05	0, 0-0.41	0, 0-0.23	0, 0-0.36	5.68E-04	0.016
OTU0259	0, 0-0.18	0.04, 0-0.17	0, 0-0.16	0, 0-0.10	0.011	0.087
OTU0265	0, 0-0.35	0.15, 0-0.67	0, 0-0.32	0.05, 0-0.49	5.51E-04	0.381
OTU0274	0, 0-0.17	0.06, 0-0.49	0, 0-0.84	0.05, 0-0.40	0.003	0.193
OTU0276	0, 0-0.20	0, 0-0.59	0, 0-0.34	0, 0-0.34	0.007	0.155
OTU0298	0, 0-0.06	0, 0-0.33	0, 0-0.05	0, 0-0.05	0.011	0.531
OTU0307	0, 0-0.78	0.11, 0-1.63	0, 0-0.64	0.08, 0-1.12	0.001	0.079
OTU0366	0, 0-0.96	0.16, 0-0.72	0, 0-0.24	0, 0-0.20	4.66E-04	0.433
OTU0475	0, 0-0.06	0, 0-0.13	0, 0-0.09	0, 0-0.07	0.022	0.169
OTU0489	0, 0-0.27	0, 0-0.16	0, 0-0.13	0, 0-0.11	0.001	0.584
OTU0160	0, 0-0.20	0.09, 0-1.26	0, 0-0.14	0, 0-0.09	6.53E-05	0.844
OTU0236	0, 0-0.05	0, 0-0.28	0, 0-0.06	0, 0-0.11	0.003	0.900
OTU0174	0.47, 0-2.85	0.14, 0-3.20	0, 0-0.21	0, 0-0.07	0.003	0.101
OTU0048	0.61, 0.18-2.66	0.40, 0-2.53	0, 0-0.33	0.05, 0-0.48	0.084	0.011
OTU0186	0.13, 0-0.53	0.05, 0-0.29	0.10, 0-0.85	0.02, 0-0.48	0.009	0.103
OTU0392	0.16, 0-1.11	0.05, 0-0.29	0.05, 0-0.47	0.02, 0-0.76	6.82E-05	0.466
OTU0153	0.09, 0-0.91	0.04, 0-0.46	0, 0-1.25	0, 0-0.26	0.037	0.642
OTU0285	0, 0-0.22	0, 0-0.06	0, 0-0.42	0.10, 0-0.31	0.232	0.004
OTU0305	0, 0-0.37	0, 0-0.65	0, 0-0.13	0, 0-0.25	0.015	0.015
OTU0581	0, 0-0	0, 0-0	0, 0-0.05	0, 0-0.17	1.000	0.025
OTU0415	0, 0-0	0, 0-0.14	0, 0-0.21	0, 0-1.03	0.011	0.059
OTU0171	0, 0-0.18	0, 0-0.16	0, 0-0.18	0, 0-0.68	0.030	0.016
OTU0249	0, 0-0.37	0, 0-0.68	0, 0-0.00	0, 0-1.27	0.175	0.001
OTU0556	0, 0-0	0, 0-0	0, 0-0.05	0, 0-0.56	1.000	0.011
OTU0173	0.04, 0-1.42	0.17, 0-2.45	0.25, 0-6.81	0.65, 0-11.1	0.031	0.019
OTU0334	0, 0-0.14	0, 0-0.33	0.04, 0-0.64	0.18, 0-0.69	0.050	9.00E-04
OTU0615	0, 0-0.11	0, 0-0.14	0.05, 0-0.24	0.13, 0-0.73	0.041	0.006
OTU0108	3.71, 0-15.3	2.69, 0.20-19.8	0.13, 0-1.46	0.47, 0-3.17	0.690	3.20E-04
OTU0179	0.36, 0-7.31	0.76, 0-12.8	0.02, 0-2.80	0.55, 0-10.2	0.525	1.66E-05
OTU0193	0.17, 0-6.22	0.45, 0-3.89	0.11, 0-1.86	0.55, 0-14.2	0.072	0.007
OTU0228	0, 0-0.84	0.05, 0-0.96	0, 0-0.16	0.08, 0-1.35	0.144	3.18E-04
OTU0358	0, 0-0.24	0.05, 0-0.62	0.06, 0-0.90	0.24, 0-2.04	0.101	0.026
OTU0341	0.07, 0-0.62	0.04, 0-1.27	0, 0-0.05	0, 0-0.47	0.324	0.007
OTU0510	0, 0-0	0, 0-0	0, 0-0	0, 0-0.17	1.000	6.33E-05
OTU0492	0, 0-0	0, 0-0.05	0, 0-0	0, 0-0.07	0.040	0.021
OTU0032	0.30, 0-2.26	0.33, 0-5.85	0.05, 0-0.80	0.12, 0-2.08	0.935	0.015
OTU0088	0.10, 0-0.97	0.13, 0-3.26	0.08, 0-1.66	0.28, 0.04-1.61	0.203	3.20E-04
OTU0350	0, 0-0.18	0, 0-0.28	0, 0-0.11	0, 0-0.24	0.105	0.007
OTU0264	0, 0-0.40	0, 0-0.19	0, 0-0	0, 0-0.10	0.830	0.021
OTU0074	0.25, 0-3.61	0.26, 0-2.60	0.26, 0-1.81	0.69, 0.15-2.75	0.668	7.90E-05
OTU0072	0.08, 0-1.51	0.20, 0-1.87	0.63, 0-2.73	1.29, 0.42-3.24	0.004	1.37E-04
OTU0437	0, 0-0	0, 0-0.25	0, 0-0.21	0.04, 0-0.34	0.021	0.006

OTU0402	0, 0-0.09	0, 0-0.08	0, 0-0	0, 0-0.15	0.688	0.040
OTU0138	1.29, 0.19-3.13	0.95, 0-4.39	0.11, 0-1.57	0.46, 0-2.39	0.322	7.75E-04
OTU0539	0, 0-0.06	0, 0-0.06	0, 0-0.09	0, 0-0.28	0.557	0.022
OTU0503	0, 0-0	0, 0-0.05	0, 0-0.04	0, 0-0.10	0.154	4.88E-04
OTU0197	0.06, 0-2.46	0.14, 0-1.59	0.42, 0-4.00	1.26, 0-5.29	0.365	0.004
OTU0270	0, 0-0.27	0, 0-0.47	0, 0-0.72	0.16, 0-2.08	0.083	0.009
OTU1609	0, 0-0.14	0, 0-0.06	0, 0-0.14	0, 0-0.11	0.097	0.007
OTU0424	0, 0-0.18	0, 0-0.53	0, 0-0.13	0, 0-0.33	0.070	0.010
OTU0183	0.07, 0-0.37	0.16, 0-0.51	0.11, 0-0.66	0.19, 0-0.69	0.003	0.042
OTU0313	0, 0-0.18	0.04, 0-0.28	0.05, 0-0.66	0.23, 0-0.78	0.021	6.44E-04
OTU0364	0, 0-0.13	0, 0-0.29	0.05, 0-0.71	0.18, 0-0.60	0.169	0.005
OTU0423	0, 0-0.44	0.04, 0-0.33	0.05, 0-0.63	0.13, 0-0.58	0.080	0.029
OTU0058	0, 0-0.73	0.09, 0-2.22	0.04, 0-1.56	0.31, 0-1.31	0.017	0.010
OTU0151	0, 0-0.99	0.07, 0-0.60	0.05, 0-1.03	0.13, 0-0.94	0.036	0.003
OTU0226	0, 0-0.38	0.02, 0-0.37	0, 0-0.32	0.07, 0-0.34	0.031	0.006
OTU0210	0.08, 0-0.55	0.06, 0-1.66	0.17, 0-2.42	0, 0-1.39	0.631	0.005
OTU0107	0.52, 0-4.98	0.44, 0-4.67	0.66, 0-9.40	0.14, 0-1.60	0.723	7.80E-05
OTU0162	4.59, 0.78-34.1	4.22, 0-18.1	2.37, 0.05-16.1	0.75, 0-6.17	0.359	8.77E-04
OTU0201	0.79, 0.06-2.82	0.40, 0-1.90	5.90, 0.94-14.3	3.04, 0.87-14.9	0.007	7.49E-04
OTU0181	0.33, 0-5.08	0.24, 0-1.99	0.77, 0.05-6.61	0.23, 0-1.87	0.700	5.10E-04
OTU0242	0.06, 0-1.84	0.02, 0-2.30	0.32, 0-8.75	0.05, 0-3.68	0.821	0.016
OTU0992	0, 0-0.25	0, 0-0.10	0, 0-0.19	0, 0-0.07	0.607	0.006
OTU1199	0, 0-0.13	0, 0-0.21	0.05, 0-0.24	0, 0-0.10	0.683	8.19E-04
OTU0199	0.35, 0-4.15	0.16, 0-1.40	1.89, 0.31-8.71	0.97, 0.25-2.83	0.036	0.003

Enriched in periodontitis patients

Enriched in healthy controls

Supplementary Table S6 Distributions of the 21 key OTUs in subgingival plaque of periodontitis.

OTU name	Relative contribution (%) [1]	Relative abundance (%) (median, range)	Prevalence (%)
**OTU0054**	18.70	20.7, 0-47.2	93.3
**OTU0096**	3.51	3.05, 0-13.1	96.7
**OTU0204**	2.97	2.51, 0-9.97	93.3
**OTU0263**	2.05	1.96, 0.10-4.96	100.0
**OTU0308**	0.74	0.65, 0-1.92	93.3
**OTU0223**	0.69	0.57, 0.05-1.99	100.0
**OTU0115**	0.50	0.30, 0-2.02	96.7
**OTU0290**	0.35	0.21, 0-2.41	66.7
**OTU0287**	0.33	0.22, 0-1.86	76.7
**OTU0304**	0.32	0.23, 0-1.06	90.0
**OTU0200**	0.32	0.15, 0-3.30	76.7
**OTU0413**	0.27	0.20, 0-0.83	93.3
**OTU0211**	0.24	0.21, 0-0.89	83.3
**OTU0177**	0.19	0.10, 0-0.73	83.3
**OTU0198**	0.13	0.05, 0-0.76	53.3
**OTU0312**	0.12	0, 0-0.65	40.0
**OTU0456**	0.11	0.06, 0-0.57	63.3
**OTU0146**	0.09	0, 0-1.25	30.0
**OTU0089**	0.08	0.08, 0-0.25	63.3
**OTU0463**	0.07	0, 0-0.37	36.7
**OTU0309**	0.03	0, 0-0.16	43.3
**Total**	31.80	/	/

[1] Relative contribution of an OTU in subgingival plaque of patients was calculated as percentage of the reads of this OTU to all reads in P-Sub group.

Supplementary Table S7 Diagnostic values of the 21 periodontitis-enriched OTUs and *F. alocis*-centered co-occurrence group.

Habitat	OTU number	Area under ROC curve	Cut-off point (%)	Sensitivity	Specificity	Likelihood ratio[1]	P value[1]
**Saliva**	OTU0263	0.786	0.32	0.73	0.80	18.1	2.11E-05
	OTU0204	0.748	0.10	0.83	0.60	12.5	4.14E-04
	OTU0308	0.777	0.04	0.80	0.70	15.9	6.68E-05
	OTU0177	0.776	0.02	0.73	0.80	18.1	2.11E-05
	OTU0054	0.769	0.37	0.77	0.73	15.7	7.35E-05
	OTU0413	0.748	0.04	0.67	0.83	16.3	5.47E-05
	OTU0096	0.783	0.76	0.77	0.77	18.0	2.23E-05
	OTU0198	0.677	0.02	0.47	0.87	8.3	3.99E-03
	OTU0287	0.742	0.02	0.57	0.90	15.8	6.96E-05
	OTU0200	0.637	0.12	0.57	0.80	8.8	3.01E-03
	OTU0223	0.669	0.02	0.57	0.77	7.1	7.66E-03
	OTU0304	0.723	0.07	0.50	0.93	15.2	9.45E-05
	OTU0089	0.726	0.02	0.60	0.80	10.4	1.29E-03
	OTU0312	0.633	0.02	0.27	1.00	12.3	4.47E-04
	OTU0463	0.617	0.02	0.23	1.00	10.6	1.11E-03
	OTU0115	0.753	0.56	0.73	0.73	13.6	2.28E-04
	OTU0290	0.687	0.02	0.47	0.90	10.6	1.15E-03
	OTU0211	0.767	0.07	0.60	0.83	12.5	4.14E-04
	OTU0456	0.637	0.02	0.30	0.97	8.6	3.28E-03
	OTU0146	0.690	0.13	0.40	1.00	19.7	9.22E-06
	OTU0309	0.633	0.02	0.27	1.00	12.3	4.47E-04
	F. alocis-centered co-occurrence group	0.817	2.11	0.87	0.73	23.8	1.10E-06
**Supragingival Plaque**	OTU0263	0.703	0.15	0.60	0.77	8.5	3.50E-03
	OTU0204	0.677	0.15	0.67	0.67	6.8	9.14E-03
	OTU0308	0.719	0.05	0.67	0.73	9.9	1.63E-03
	OTU0177	0.629	0.05	0.40	0.90	7.6	5.85E-03
	OTU0054	0.752	0.36	0.87	0.57	13.1	2.99E-04
	OTU0413	0.650	0.02	0.57	0.73	5.7	1.74E-02
	OTU0096	0.686	0.44	0.67	0.77	11.8	5.96E-04
	OTU0198	0.683	0.02	0.37	1.00	17.7	2.53E-05
	OTU0287	0.679	0.07	0.43	0.93	11.7	6.15E-04
	OTU0200	0.691	0.02	0.63	0.77	10.1	1.50E-03
	OTU0223	0.666	0.05	0.73	0.60	6.9	8.47E-03
	OTU0304	0.708	0.10	0.43	0.97	15.4	8.84E-05
	OTU0089	0.687	0.02	0.43	0.93	11.7	6.15E-04
	OTU0312	0.583	0.02	0.17	1.00	7.4	6.57E-03
	OTU0463	0.567	0.02	0.13	1.00	5.8	1.57E-02
	OTU0115	0.710	0.15	0.70	0.73	11.7	6.37E-04
	OTU0290	0.690	0.10	0.47	0.93	13.4	2.46E-04
	OTU0211	0.794	0.10	0.83	0.73	20.7	5.24E-06
	OTU0456	0.676	0.05	0.70	0.70	9.9	1.68E-03
	OTU0146	0.601	0.02	0.23	0.97	5.8	1.64E-02
	OTU0309	0.600	0.02	0.20	1.00	9.0	2.72E-03
	F. alocis-centered co-occurrence group	0.739	1.58	0.77	0.63	10.1	1.50E-03

[1] Likelihood ratio and P values were assessed by Chi-Square test

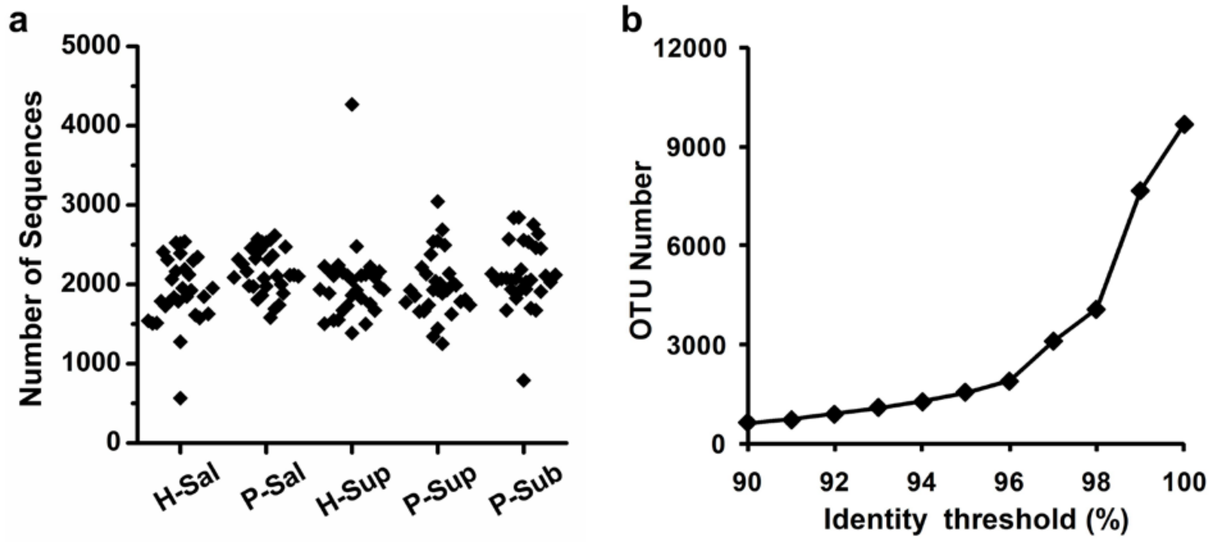

Supplementary Figure S1 (a) The number of sequences for each sample. **(b)** The numbers of OTUs identified at several different similarity levels. The x-axis shows the identity, the y-axis shows the number of OTUs. OTUs were delineated at 96% similarity level because higher thresholds generated a dramatic increase of OTU numbers, which might represent the microdiversity at subspecies level.

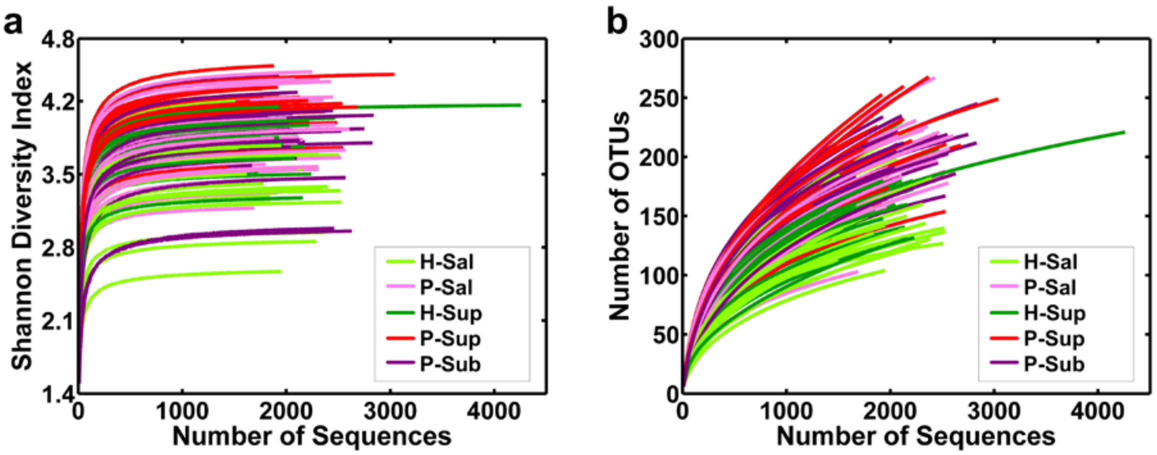

Supplementary Figure S2 Shannon diversity index curves **(a)** and rarefaction curves **(b)** of oral microbiota in five groups.

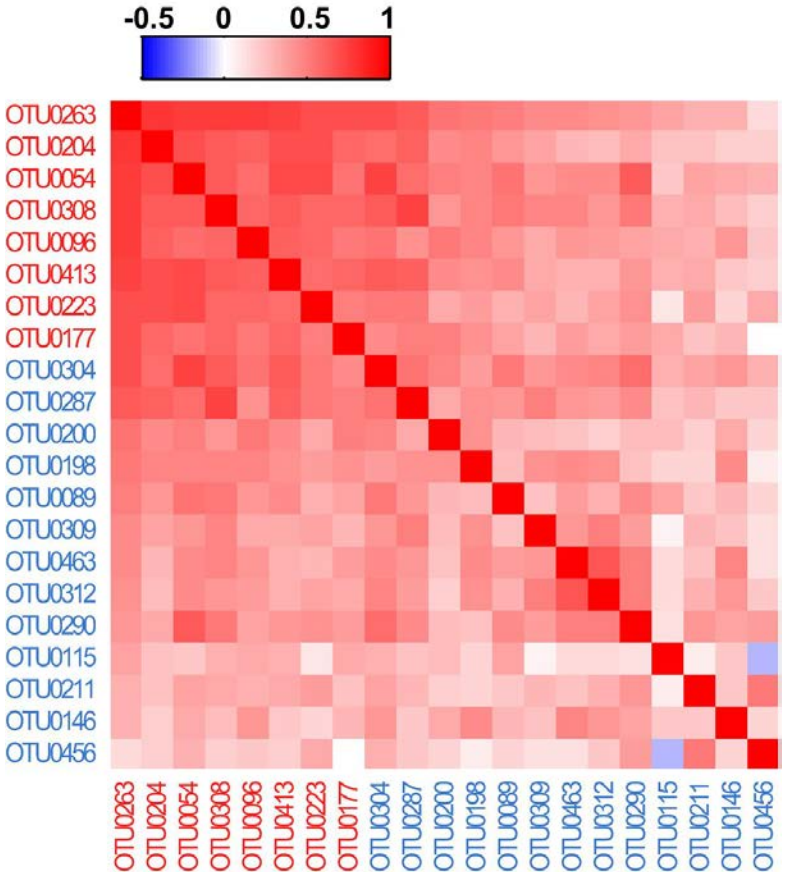

Supplementary Figure S3 Heat map of the correlation between 21 key OTUs in the oral microbiota. The color of the spot corresponds to the pairwise Spearman's correlation coefficient of each two OTUs calculated based on their normalized relative abundances in all 150 samples. The eight members of *F. alocis*-centered co-occurrence group were colored in red, and others were colored in blue.
